# Salicylate induces AMPK and inhibits c-*MYC* to activate a NRF2/ARE/*miR-34a/b/c* cascade resulting in suppression of colorectal cancer metastasis

**DOI:** 10.1038/s41419-023-06226-9

**Published:** 2023-10-28

**Authors:** Chunfeng Liu, Matjaz Rokavec, Zekai Huang, Heiko Hermeking

**Affiliations:** 1 Experimental and Molecular Pathology, Institute of Pathology, Faculty of Medicine, Ludwig-Maximilians-Universität München, Thalkirchner Strasse 36, D-80337 Munich, Germany; 2 German Cancer Consortium (DKTK), Partner site Munich, D-80336 Munich, Germany; 3 German Cancer Research Center (DKFZ), D-69210 Heidelberg, Germany

**Keywords:** Colon cancer, Translational research

## Abstract

Aspirin and its active metabolite salicylate have emerged as promising agents for the chemoprevention of colorectal cancer (CRC). Moreover, aspirin suppresses the progression of established CRCs. However, the underlying molecular mechanisms are not completely understood. Here we found that salicylate induces the expression of the *miR-34a* and *miR-34b/c* genes, which encode tumor suppressive microRNAs, in a p53-independent manner. Salicylate activated AMPK, thereby activating NRF2, which directly induced *miR-34a/b/c* expression via ARE motifs. In addition, salicylate suppressed c-MYC, a known repressor of NRF2-mediated transactivation, via activating AMPK. The suppression of c-MYC by salicylate was necessary for NRF2-mediated activation of *miR-34a/b/c*. Inactivation of miR-34a/b/c largely abrogated the inhibitory effects of salicylate on migration, invasion and metastasis formation by CRC cells. In the future, aspirin and its derivates may be used therapeutically to activate *miR-34a* and *miR-34b/c* in tumors that have lost p53.

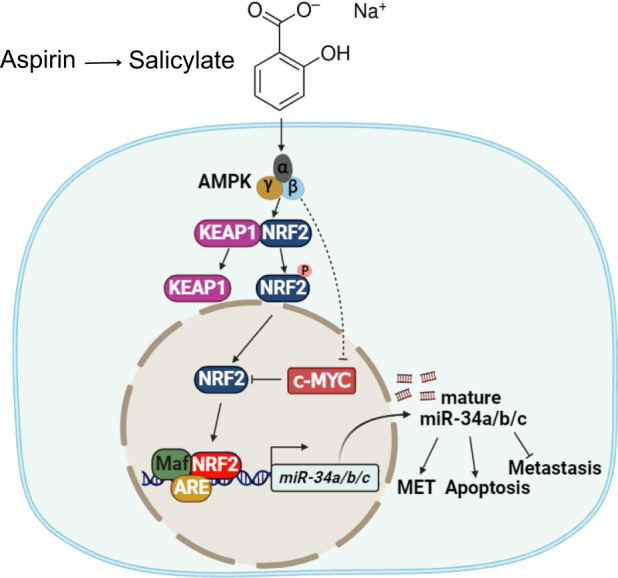

## Introduction

Colorectal cancer (CRC) is the third most commonly diagnosed malignancy with more than 1.9 million annual diagnoses worldwide and the second leading cause of cancer mortality, responsible for more than 900,000 deaths [1]. Colonoscopy-based screening strategies have demonstrated their potential for decreasing the incidence and mortality of CRC. Another strategy to reduce CRC occurrence is the use of chemo-preventive drugs. Among them, aspirin is perhaps the most promising substance [2].

Aspirin, also known as acetylsalicylic acid, is a nonsteroidal anti-inflammatory drug primarily known for its analgesic and antipyretic actions [3]. However, several decades of research also provided considerable evidence demonstrating its potential for the prevention of cancer, particularly CRC [2]. A pooled analysis of 10 case–control studies with more than 8000 CRC cases demonstrated that intake of aspirin was associated with a 29% reduction in the incidence of CRC (odds ratio (OR) = 0.71; 95% confidence interval (CI) 0.66–0.77) [4]. Another case-control meta-analysis of 17 studies with more than 12000 CRC cases showed that regular use of aspirin was associated with reduced risk for CRC (OR = 0.52, 95% CI 0.58–0.67). Furthermore, a meta-analysis of four randomized controlled trials with more than 14000 CRC patients found that aspirin treatment for 5 or more years at doses of 75–300 mg per day reduced the long-term risk of CRC by 24% (hazard ratio (HR) = 0.75; 95% CI 0.56–0.97) [5]. In 2015 the United States Preventive Services Task Force endorsed aspirin as the first pharmacological agent for cancer chemoprevention in a population not characterized as having a high risk of developing cancer [2]. In addition to cancer prevention, several studies have shown that aspirin use after CRC diagnosis was associated with reduced mortality [3]. A recent meta-analysis of 27 studies with more than 230,000 CRC patients showed that aspirin use after diagnosis was associated with an improvement in CRC-specific survival (HR = 0.74 (95% CI: 0.62–0.89) and overall survival (HR = 0.82 (95% CI: 0.74–0.90) [6]. CRC patients treated with a daily low-dose aspirin are less likely to develop advanced stage CRC, suggesting that aspirin affects the progression of established CRCs [7]. Following oral consumption, aspirin is absorbed mainly in the stomach and upper small intestine [8]. The half-life of aspirin is about 20 minutes as it undergoes hydrolysis to generate salicylic acid and salicylate [8, 9].

The molecular mechanisms by which aspirin decreases CRC initiation and progression are incompletely understood. Aspirin acts directly on CRC cells and indirectly via affecting the tumor microenvironment. Aspirin inhibits COX enzymes in epithelial and stromal cells, which reduces the synthesis of prostaglandins [10]. Aspirin also inhibits the Wnt/β-catenin pathway, which is the oncogenic driver in the majority of CRCs [11]. Activated Wnt signaling induces the expression of the oncogene c-*MYC*. Consistently, it has been shown that aspirin down-regulates c-MYC levels in numerous cancer entities [12, 13].

*miR-34a* and *miR-34b/c* represent p53-inducible genes, which encode microRNAs with tumor-suppressive properties [14, 15]. They are frequently down-regulated in human CRCs by methylation [16]. The loss of *miR-34a* promotes CRC development in mouse models of sporadic CRC (6xAOM treatment [17]), colitis-associated CRC (AOM/DSS treatment [18]), and inherited CRC (*Apc*^*Min/+*^ mice [19]). Therefore, down-regulation of *miR-34a* does not only commonly occur during intestinal carcinogenesis, but is also causally involved in CRC formation.

Here we hypothesized that miR-34a and miR-34b/c may represent mediators of the suppressive effects of aspirin on CRC cells. Indeed, we found, that salicylate, the main metabolite of aspirin, induces the expression of *miR-34a* and *miR-34b/c* in a p53-independent manner via activating of AMP-activated protein kinase (AMPK), which activates nuclear factor erythroid 2–related factor 2 (NRF2). Furthermore, we demonstrated that the suppression of c-MYC by salicylate is required for NRF2-mediated activation of *miR-34a* expression. The induction of *miR-34a* was necessary for multiple tumor suppressive effects of salicylate, including the repression of cell viability, migration, invasion, and metastasis, as well as for the induction of apoptosis and mesenchymal-to-epithelial transition (MET). In the future, this knowledge may be used to activate miR-34 function in tumors, which have lost p53 function.

## Results

### Salicylate inhibits cell viability and induces apoptosis in CRC cells

**Fig. 1:**
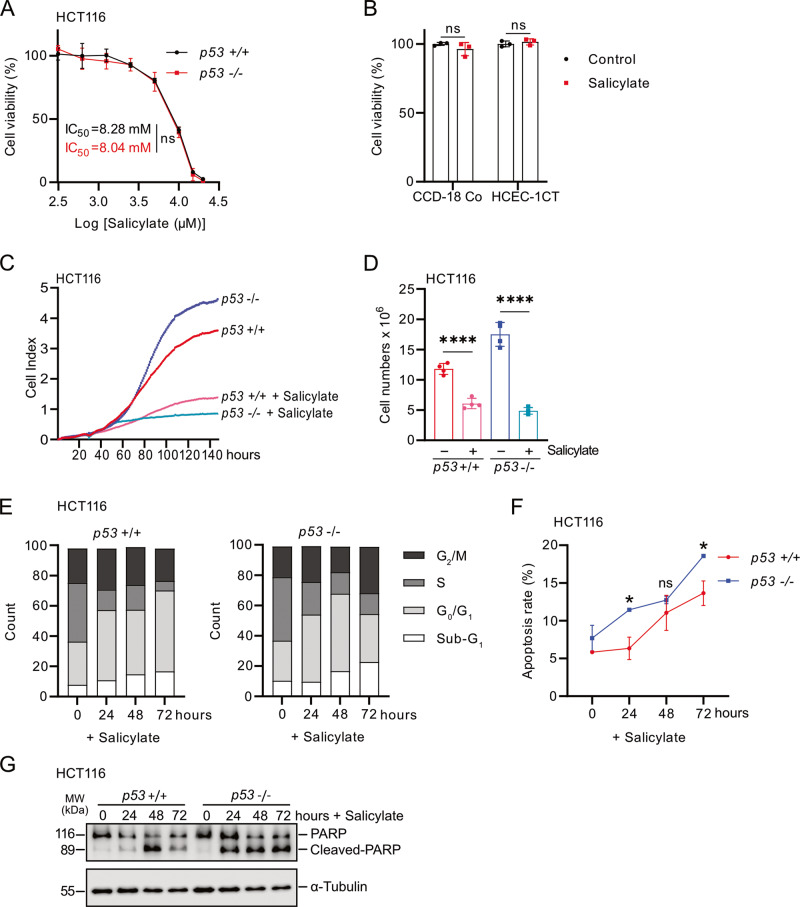
Salicylate suppresses viability and proliferation of CRC cells independent of p53. **A** Cell viability of HCT116 cells was determined by MTT assay after treatment with indicated concentrations of salicylate for 48 h. IC_50_ was determined using GraphPad Prism based on changes in viability. **B** Cell viability of non-transformed CCD-18-Co and HCEC-1CT cells was determined by MTT assay after treatment with 5 mM salicylate for 48 h. **C** Impedance of HCT116 cells treated with salicylate. **D** Determination of cell numbers at the final time point of the experiment indicated in **C**. **E** Cell cycle analysis using propidium iodide (PI) staining. **F** Analysis of apoptosis in salicylate-treated HCT116 cells by Annexin V FITC and propidium iodide staining. **G** Levels of cleaved PARP and PARP were determined by Western blot analysis in HCT116 cells after treatment with 5 mM salicylate for the indicated times. α-Tubulin served as a loading control. In panels **A**, **B**, and **F** (*n* = 3), and **D** (*n* = 4) mean values ± SD are shown. *p < 0.05, ****p < 0.0001, n.s not significant.

It has been previously shown that salicylate, which is the active metabolite of aspirin, represses the viability of CRC cells [20]. In order to systematically investigate whether p53 mediates the effect of salicylate on CRC cells, we treated HCT116 cells harboring wild-type *p53* and isogenic HCT116 cells with homozygous deletion of *p53* with increasing concentrations of salicylate for 48 h. *p53*-proficient and *p53*-deficient HCT116 cells displayed similar IC_50_ values (Fig. 1A). Based on the IC_50_ values and previous publications [21–24], in which CRC cells are treated with salicylate, a concentration of 5 mM was chosen for the subsequent experiments. Notably, this concentration of salicylate did not show obvious cytotoxicity in the non-transformed human colonic epithelial cells (HCEC-1CT) and human intestinal fibroblasts (CCD-18Co) (Fig. 1B), indicating that non-transformed epithelial cells and fibroblasts in the intestine may be resistant to the effects of salicylate. In contrast, salicylate strongly inhibited proliferation of *p53*-proficient and *p53*-deficient HCT116 CRC cells as determined by impedance measurements (Fig. 1C). The changes in cell numbers were confirmed at the final time point (Fig. 1D). Salicylate treatment resulted in an increase of cells in the G_0_/G_1_-phases and a decrease of cells in S- and G_2_/M-phases, irrespective of their *p53* status (Fig. 1E and Supplementary Fig. S1A). The increase of cells in the sub-G_1_-phase indicated that salicylate induced apoptosis, which was more prominent in *p53*-deficient than in *p53*-proficient HCT116 cells as determined by flow-cytometric analysis of Annexin V/PI staining (Fig. 1F and Supplementary Fig. S1B). These results were confirmed by detection of cleaved-PARP protein levels (Fig. 1G). Similar effects were observed in *p53*-proficient or *p53*-deficient RKO CRC cell lines (Supplementary Fig. S1C). Altogether, these results demonstrate that salicylate represses proliferation and induces apoptosis in a *p53*-independent manner in CRC cells.

### Salicylate inhibits migration, invasion and induces MET in CRC cells

**Fig. 2:**
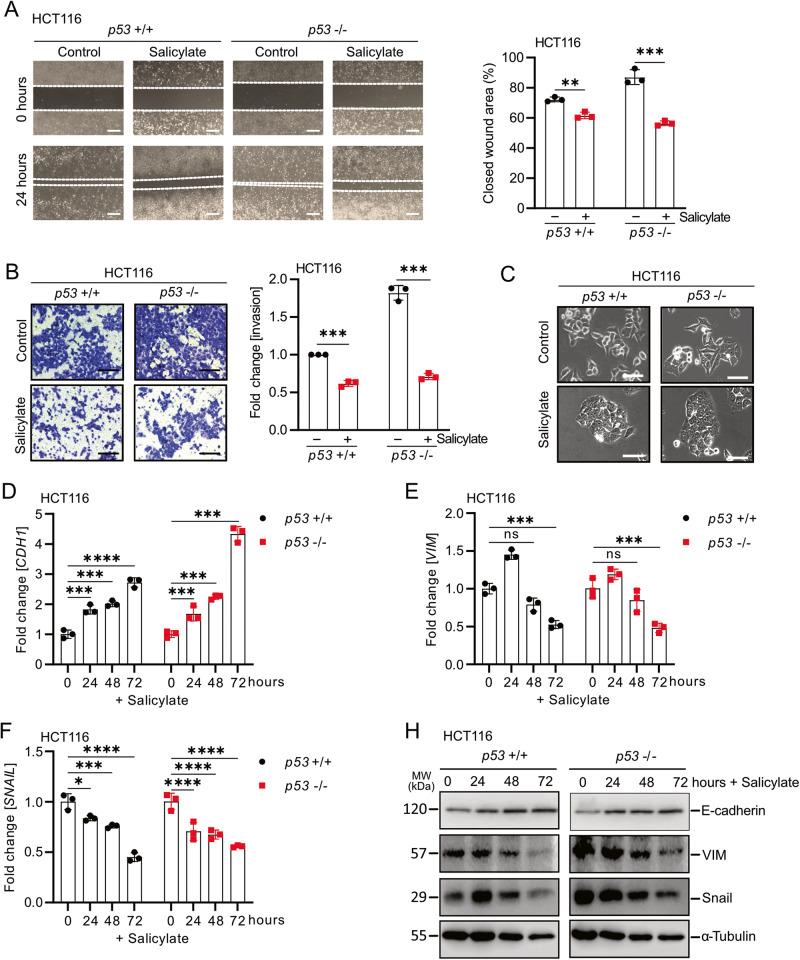
Salicylate inhibits migration, invasion and induces MET independent of p53. **A** Wound healing assay. The scratch width was determined 24 h after the indicated treatment (left panel). Results represent the mean (%) of wound closure (right panel). **B** Determination of invasion by a Modified Boyden chamber assay. After treatment for 48 h, cells that invaded through the matrigel were counted after crystal violet staining. **C** Phase-contrast images showing cell morphology of HCT116 cells treated with 5 mM salicylate for 48 h. Scale bar: 50 μm. qPCR analysis of *CDH1* (**D**), *VIM* (**E**), and *SNAIL* (**F**) expression in HCT116 cells after the indicated treatments for 48 h. **H** Western blot analysis of protein expression after salicylate treatment for the indicated periods. In panels **A**–**F** (*n* = 3), mean values ± SD are shown. *p < 0.05, **p < 0.01, ***p < 0.001, ****p < 0.0001.

Next, we evaluated the effects of salicylate on cell migration and invasion. Salicylate repressed cell migration and invasion in *p53*-proficient and *p53*-deficient HCT116 cells (Fig. 2A, B). Similar results were observed in *p53*-proficient and *p53*-deficient RKO CRC cell lines (Supplementary Fig. S2A, S2B). Salicylate treatment resulted in a switch from a mesenchymal to an epithelial morphology in *p53*-proficient and *p53*-deficient HCT116 cells (Fig. 2C), indicating a mesenchymal-to-epithelial transition (MET). Indeed, treatment of *p53*-proficient or *p53*-deficient HCT116 cells with salicylate resulted in the repression of the mesenchymal markers Vimentin (VIM) and SNAIL, while the expression of the epithelial marker E-cadherin/CDH1 was increased on mRNA and protein levels (Fig. 2D–H). Similar results were observed in *p53*-proficient or *p53*-deficient RKO CRC cell lines (Supplementary Fig. S2C–S2E). Taken together, these results show that salicylate suppresses migration and invasion, and induces MET in CRC cells in a *p53*-independent manner.

### Salicylate up-regulates *miR-34a* and *miR-34b/c* independent of p53

**Fig. 3:**
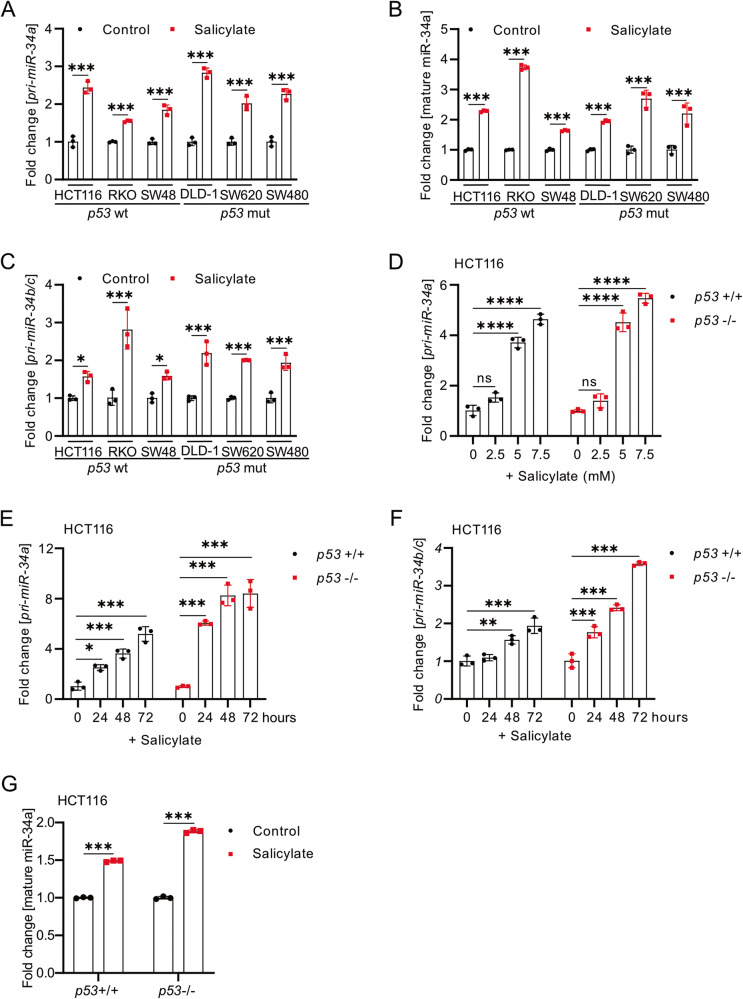
Salicylate up-regulates *miR-34a* and *miR-34b/c* independent of p53. qPCR analysis of *pri-miR-34a* (**A**), mature miR-34a (**B**), and *pri-miR-34b/c* (**C**) expression after treatment of the indicated cells with salicylate for 48 h. **D** qPCR analysis of *pri-miR-34a* expression after treatment with the indicated concentration of salicylate for 48 h. qPCR analysis of *pri-miR-34a* (**E**) and *pri-miR-34b/c* (**F**) expression after treatment with salicylate for the indicated periods. **G** qPCR analysis of mature miR-34a expression after treatment with salicylate for 48 h. In panels **A**–**G** (*n* = 3), mean values ± SD are shown. *p < 0.05, **p < 0.01, ***p < 0.001, ****p < 0.0001, n.s: not significant.

It has been previously shown that miR-34a and miR-34b/c suppress cell viability, migration and invasion, and induce apoptosis and MET [25]. Therefore, *miR-34a/b/c* represent candidate mediators of the effects of salicylate and we analyzed whether they are induced by salicylate. Indeed, treatment with salicylate induced the expression of *pri-miR-34a*, mature miR-34a, and *pri-miR-34b/c* in three CRC cell lines with wildtype *p53* and also in three *p53* mutant CRC cell lines (Fig. 3A–C). Furthermore, salicylate induced the expression of primary *pri-miR-34a* and *pri-miR-34b/c* in a dose- and time-dependent manner in HCT116 cells with deletion of *p53* in a similar manner as in HCT116 wt *p53* cells (Fig. 3D–F). Similar results were obtained in wt and KO *p53* RKO cells (Supplementary Fig. S3A, B). The levels of mature miR-34a were also up-regulated by salicylate in *p53*-deficient HCT116 and RKO cells (Fig. 3G and Supplementary Fig. S3C). Therefore, salicylate induces *miR-34a* and *miR-34b/c* expression in CRC cell lines in a *p53*-independent manner.

### *miR-34a* and *miR-34b/c* mediate the effects of salicylate in CRC cells

**Fig. 4:**
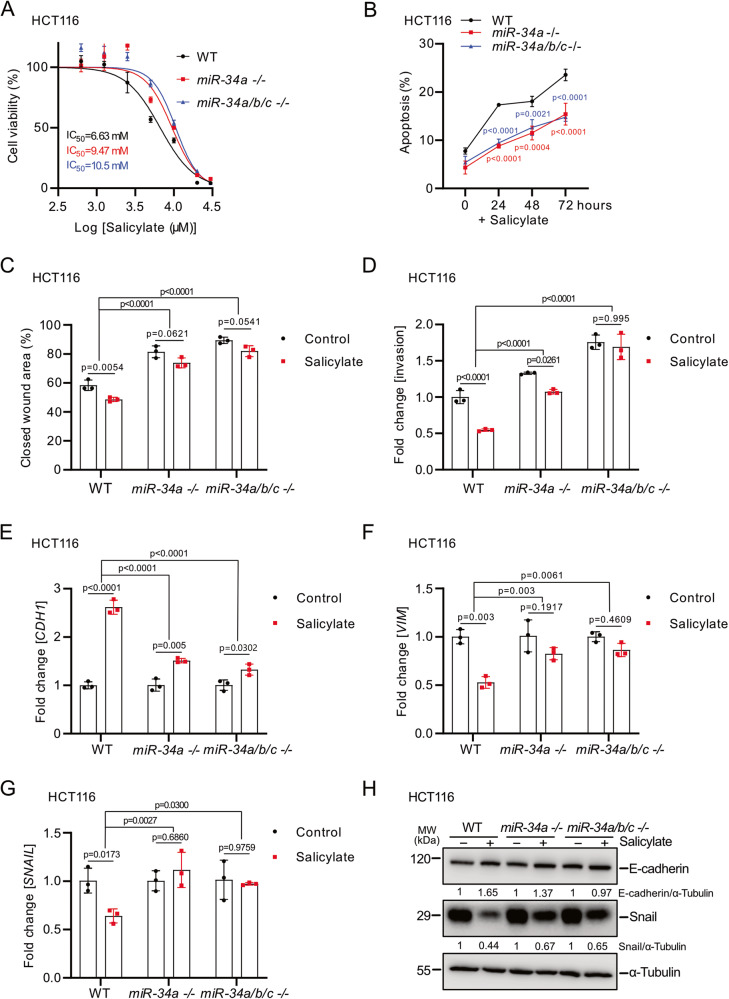
*miR-34a* and *miR-34-b/c* mediate the effects of salicylate in CRC cells. **A** Treatment of the indicated CRC cell lines with increasing concentrations of salicylate for 48 h. IC_50_ was determined by MTT assay. **B** After treatment with salicylate for the indicated periods apoptosis was determined by quantification of Annexin V-FITC and PI staining. **C** Migration was assessed by wound-healing assay 24 h after salicylate treatment. **D** Invasion was analyzed using a modified Boyden chamber assay 48 h after salicylate treatment. qPCR analysis of *CDH1* (**E**), *VIM* (**F**), and *SNAIL* (**G**) expression in HCT116 cells after the indicated treatments for 48 h. **H** Western blot analysis of E-cadherin and SNAIL protein levels after salicylate treatment for the indicated periods. In panels **A**–**G** (*n* = 3), mean values ± SD are shown.

To determine whether the induction of *miR-34a* and *miR-34b/c* expression mediates the effects of salicylate, we analyzed isogenic wt and *miR-34a-* or *miR-34a/b/c*-deficient HCT116 cells, that were recently generated using a CRISPR/CAS9 approach and characterized by us [26]. After treatment with salicylate for 48 h the viability of *miR-34a-* or *miR-34a/b/c*-deficient HCT116 cells was higher than that of *miR-34*-proficient HCT116 cells (Fig. 4A). Consistently, *miR-34a-* or *miR-34a/b/c*-deficiency resulted in an attenuated induction of apoptosis by salicylate in HCT116 cells (Fig. 4B and Supplementary Fig. S4A). Furthermore, inhibition of migration and invasion by salicylate was significantly lower in *miR-34a* and *miR-34a/b/c*-deficient cells when compared to *miR-34*-proficient HCT116 cells (Fig. 4C, D, Supplementary Fig. S4B, S4C). Finally, the deletion of *miR-34a* or *miR-34a/b/c* partly abolished salicylate-induced MET, as salicylate treatment resulted in attenuated induction of *CDH1* and decreased suppression of *VIM* and *SNAIL* in *miR-34a*^*−/−*^ and *miR-34a/b/c*^*−/−*^ HCT116 cells (Fig. 4E–H). Taken together, our results demonstrate that *miR-34a* and *miR-34b/c* mediate the effects of salicylate on cell viability, apoptosis, migration, invasion, and MET to a large extent.

### Salicylate inhibits lung metastases formation through induction of miR-34a

**Fig. 5:**
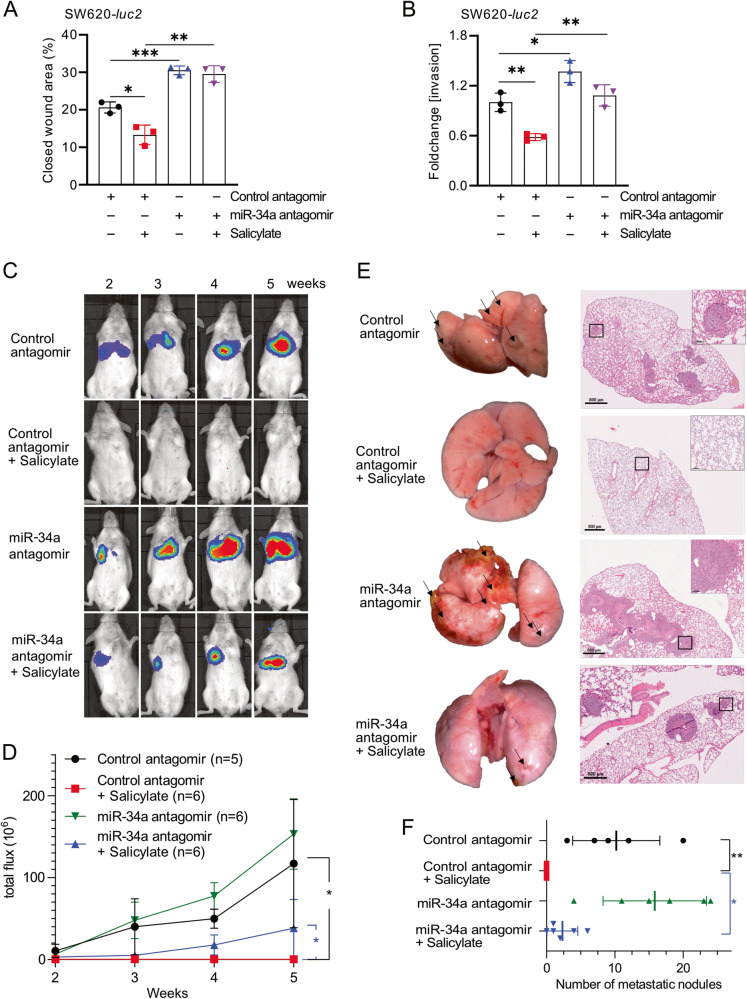
Salicylate inhibits lung metastases formation through induction of miR-34a. Analysis of migration (**A**) and invasion (**B**) after transfection of SW620-*Luc2* cells with miR-34a antagomirs and/or treatment with salicylate for 72 h. **C**–**F** SW620-*Luc2* cells were treated for 48 h as indicated and then injected into the tail vein of NOD/SCID mice. Representative images of luciferase signals (**C**) and quantification of total photon flux (**D**) at indicated time points after xenografting. **E** left: representative lungs 5 weeks after tail vein injection. Arrows indicate metastatic tumor nodules. right: representative images of H&E-stained resected lungs. Scale bar: 500 μm; 50 μm (insert). **F** Quantification of metastatic nodules in the lungs of the indicated mice. In panels **A**, **B**, **D**, and **F** (*n* = 3), mean values ± SD are shown. *p < 0.05, **p < 0.01, ***p < 0.001.

Next, we aimed to determine the effect of salicylate on metastases formation of CRC cells in mice. For this we utilized SW620 CRC cells stably expressing luciferase (SW620-*Luc2*). We have previously shown that mature miR-34b and miR-34c are expressed at very low levels in SW620-*Luc2* cells when compared to mature miR-34a [27]. Therefore, we focused our analyses on the inhibition of miR-34a. First, we confirmed that salicylate repressed migration and invasion in SW620-*Luc2* cells (Fig. 5A, B, Supplementary Fig. S5A, S5B). Notably, this effect was abolished by *miR-34a*-specific antagomirs. Next, SW620-*Luc2* cells were treated with salicylate or/and *miR-34a*-specific antagomirs for 48 h. After washing cells with HBSS for 3 times and counting the number of living cells (using trypan blue), 4 ×10^6^ cells were injected into the lateral tail vein of NOD/SCID mice. Longitudinal, non-invasive imaging revealed that treatment of SW620-*Luc2* cells with salicylate completely abolished lung metastasis formation within 5 weeks after injection (Fig. 5C, D). However, concomitant treatment with *miR-34a*-specific antagomirs partially restored metastasis formation after salicylate treatment (Fig. 5C, D). Five weeks after injection, excised lungs showed no macroscopic metastasis in mice injected with salicylate-treated SW620-*Luc2* cells (Fig. 5E). Hematoxylin and eosin (H&E) staining confirmed the absence of metastatic nodules in mice injected with salicylate-treated cells (Fig. 5E, F). However, SW620-*Luc2* cells concomitantly treated with *miR-34a*-antagonist and salicylate developed lung metastases (Fig. 5E, F). Therefore, salicylate suppresses metastases formation by inducing *miR-34a* in xenografted CRC cells.

### The induction of *miR-34a/b/c* by salicylate is mediated by NRF2

**Fig. 6:**
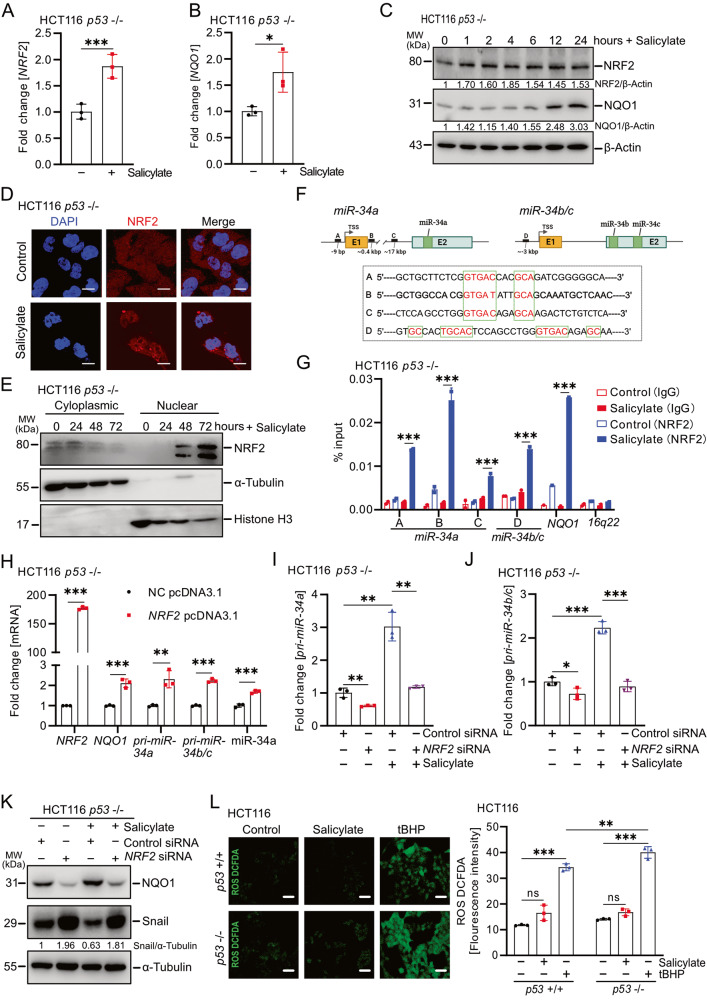
Induction of *miR-34a/b/c* by salicylate is mediated by NRF2. mRNA (**A**, **B**) and protein (**C**) levels of NRF2 and NQO1 were determined by qPCR and Western blot analysis, respectively. **D** Immunofluorescence was used to detect the location of NRF2 protein after the indicated treatments for 48 h. Nuclear DNA was detected by DAPI. The scale bar represents 20 µm. **E** Western blot analysis of NRF2 protein levels in cytoplasmic and nuclear cellular fractions after salicylate treatment. **F** Map of human *miR-34a* and *miR-34b/c* genomic regions with NRF2 binding sites A–D and respective sequences depicted. **G** qChIP analysis of NRF2 occupancy at the human *miR-34a* and *miR-34b/c* genomic regions in HCT116 *p53*−/− cells 48 h after the indicated treatments. *NQO1* and *16q22* served as positive and negative controls, respectively. **H** qPCR analysis of indicated mRNAs in *p53*-deficient HCT116 cells after transfection with empty pcDNA3.1 (NC) or *NRF2* pcDNA3.1 vectors for 72 h. qPCR analysis of *pri-miR-34a* (**I**) and *pri-miR-34b/c* (**J**) expression after indicated treatment and/or transfection with the indicated siRNA pools for 24 h in *p53*-deficient HCT116 cells. **K** Western blot analysis of NQO1 and SNAIL protein expression after treatment and/or transfection with siRNA pools for 24 h in *p53*-deficient HCT116 cells. **L** Analysis of ROS formation in HCT116 cells treated as indicated, left panel**:** representative fluorescence intensity pictures. Scale bar: 100 µm. right panel: quantification of fluorescence intensity. In panels **A**, **B**, **G**–**J**, and **L** (*n* = 3), mean values ± SD are shown. *p < 0.05, **p < 0.01, ***p < 0.001, n.s not significant.

We recently showed that curcumin induces *miR-34a* and *miR-34b/c* expression via the activation of NRF2 independent of p53 [27]. Here, salicylate also induced the expression of *NRF2* and *NQO1*, which is a conserved NRF2 target gene and can serve to monitor the activity of the NRF2 pathway (Fig. 6A–C). Furthermore, NRF2 protein accumulated and translocated to the nucleus after treatment with salicylate, confirming that salicylate activates NRF2 (Fig. 6D, E). We have previously identified three NRF2 binding sites (TGAG/CnnnGC), so-called AREs (Antioxidant Response Elements) in the *miR-34a* promoter and one in the *miR-34b/c* promoter (Fig. 6F) [27]. A qChIP assay confirmed enhanced NRF2 occupancy at these four ARE sites within the *miR-34a* and *miR-34b/c* promoters after salicylate treatment for 48 h (Fig. 6G). Ectopic *NRF2* expression increased *pri-miR-34a* and *pri-miR-34b/c* expression, as well as mature miR-34a and *NQO1* mRNA expression, in *p53*-deficient HCT116 cells (Fig. 6H). Conversely, silencing of *NRF2* by a pool of 4 different siRNAs prevented the activation of *pri-miR-34a* and *pri-miR-34b/c* after treatment with salicylate (Fig. 6I, J). The inhibitory effect of salicylate on migration, invasion, and EMT was also partly prevented after silencing *NRF2* (Supplementary Fig. S6A–S6B, Fig. 6K). We have recently reported, that curcumin activates NRF2 via generating reactive oxygen species/ROS in CRC cells [27]. However, ROS levels were not increased after treatment with salicylate (Fig. 6L). Moreover, addition of the ROS-inhibitor N-acetyl-cystein/NAC did not prevent the induction of *pri-miR-34a* and *pri-miR-34b/c* expression by salicylate in *p53*-proficient or *p53*-deficient HCT116 cells (Supplementary Fig. S6C, D). In summary, these results show that salicylate induces *miR-34a* and *miR-34b/c* expression by activating NRF2 via a ROS-independent mechanism.

### Salicylate activates NRF2 via AMPK in CRC cells

**Fig. 7:**
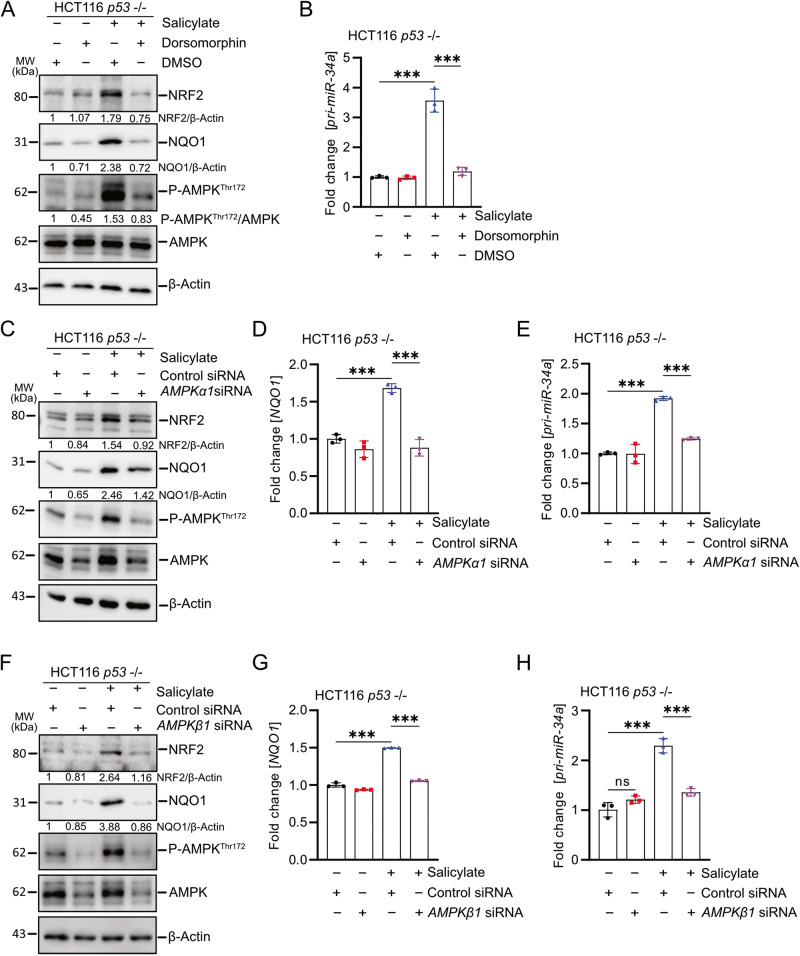
Salicylate activates NRF2 via AMPK in CRC cells. **A** Western blot analysis of indicated proteins in *p53*-deficient HCT116 cells treated with salicylate and/or 10 μm dorsomorphin for 24 h. β-actin served as a loading control. **B** qPCR analysis of *pri-miR-34a* expression after treatment with salicylate and/or 10 μm dorsomorphin for 24 h. **C** Western blot analysis of indicated proteins 48 h after transfection with *AMPK α1* siRNA or control siRNA pools with or without salicylate for the last 24 h. β-actin served as a loading control. qPCR analysis of *NQO1* (**D**) and *pri-miR-34a* (**E**) expression 48 h after transfection with *AMPK α1*-specific siRNA pools or control siRNA pools with or without salicylate for the last 24 h. **F** Western blot analysis of indicated proteins after transfection with *AMPK β1*-specific siRNA or control siRNA pools for 48 h with or without salicylate for 24 h. β-actin served as a loading control. qPCR analysis of *NQO1* (**G**) and *pri-miR-34a* (**H**) expression after indicated treatments with siRNA-pools and/or salicylate for 24 h. In panels **B**, **D**, **E**, **G**, and **H** (*n* = 3), mean values ± SD are shown. ***p < 0.001, n.s not significant.

Next, we studied alternative modes of NRF2 activation, that would explain how salicylate induces *miR-34* expression. Previously, AMPK was reported to phosphorylate and activate NRF2 [28]. Interestingly, salicylate directly binds to AMPK in the cleft between the β-CBM and the α-KD N-lobe, which causes both allosteric activation and protection against Thr172 dephosphorylation of AMPK [29]. Here, salicylate activated AMPK, which was largely prevented by the AMPK inhibitor dorsomorphin (Fig. 7A). Furthermore, the induction of NRF2 and NQO1, which is encoded by a NRF2 target gene, by salicylate was largely prevented by dorsomorphin (Fig. 7A). Finally, the induction of *pri-miR-34a* by salicylate was completely abolished by dorsomorphin (Fig. 7B). Also, suppression of *AMPKα1* by siRNA pools attenuated the induction of NRF2, NQO1, *NQO1* mRNA, and *pri-miR-34a* by salicylate (Fig. 7C–E). Furthermore, suppression of *AMPKβ1* by siRNA pools also attenuated the induction of NRF2, NQO1 and *pri-miR-34a* by salicylate (Fig. 7F–H). Similar results were observed in RKO cells (Supplementary Fig. S7A–H). Taken together, these results demonstrate that salicylate activates NRF2 and induces miR-34a via activating AMPK in CRC cells.

### c-MYC abrogates NRF2 activation and *miR-34a/b/c* induction by salicylate

**Fig. 8:**
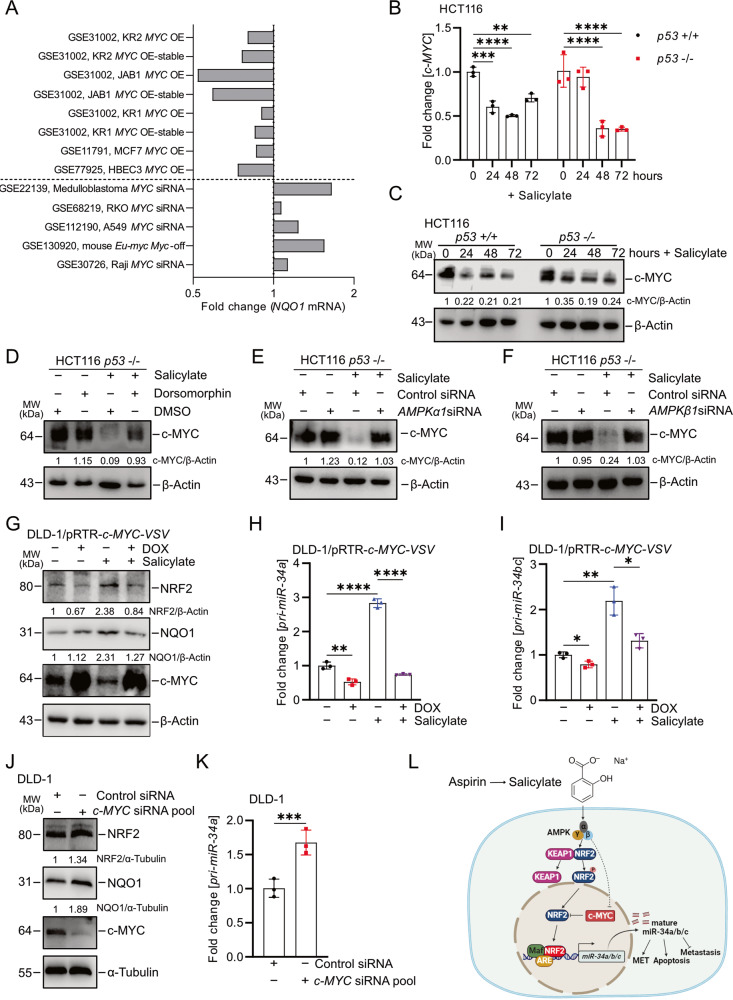
Suppression of c-*MYC* by salicylate is required for NRF2-mediated induction of *miR-34a* and *miR-34b/c*. **A** Fold changes in *NQO1* expression in GEO datasets representing studies of ectopic expression or silencing of c-*MYC* in the indicated cell lines. qPCR and Western blot analysis of c-MYC mRNA (**B**) and protein (**C**) levels after treatment with salicylate for the indicated periods. **D** Western blot analysis of c-MYC protein levels in *p53*-deficient HCT116 cells treated with salicylate and/or 10 μM dorsomorphin for 24 h. β-actin served as a loading control. Western blot analysis of indicated proteins after transfection with *AMPK α1* (**E**) or *AMPK β1* (**F**) siRNA pools or control siRNA pool for 48 h with or without salicylate for 24 h. β-actin served as a loading control. **G** Western blot analysis of c-MYC, NRF2, and NQO1 protein levels in DLD-1/*pRTR-c-MYC-VSV* cells after treatment with salicylate or/and DOX (c-MYC on) for 48 h. qPCR analyses of *pri-miR-34a* (**H**) and *pri-miR-34b/c* (**I**) expression in DLD-1/*pRTR-c-MYC-VSV* cells treated with salicylate or/and DOX (c-MYC on) for 48 h. **J** Western blot analysis of c-MYC, NRF2, and NQO1 protein levels in DLD-1 cells after transfection *c-MYC* siRNA pool or control siRNA pool for 48 h. **K** qPCR analysis of *pri-miR-34a* expression in DLD-1 cells after transfection *c-MYC siRNA* pool or control siRNA pool for 48 h. **L** Schematic model of the findings obtained in this study. In panels **B**, **H**, **I**, and **K** (*n* = 3) mean values ± SD are shown. *p < 0.05, **p < 0.01, ***p < 0.001, ****p < 0.0001.

It has been shown that salicylate suppresses c-MYC expression at both transcriptional and post-transcriptional levels [12]. Furthermore, c-MYC was shown to interact with NRF2, thereby preventing the binding of NRF2 to ARE elements and induction of NRF2 target genes [30]. Therefore, we asked whether a down-regulation of c-MYC is involved in the regulation of NRF2 activity by salicylate. First, we analyzed 13 public Gene Expression Omnibus (GEO) datasets for the effects of ectopic expression or knockdown/knockout of *c-MYC* in cell lines or mice on *NQO1* expression. Notably, the NRF2 target gene *NQO1* was repressed after ectopic expression of c-MYC and up-regulated after repression of c-MYC in the majority of these GEO studies (Fig. 8A). Treatment with salicylate repressed c-MYC at the mRNA and protein levels in a p53-independent manner (Fig. 8B, C). The suppression of c-MYC by salicylate was completely abolished by the AMPK inhibitor dorsomorphin or *AMPKα1/AMPKβ1-*specific siRNA pools (Fig. 8D–F and Supplementary Fig. S8A–C), demonstrating that salicylate suppresses the expression of c-*MYC* or the levels of c-MYC via AMPK. The induction of NRF2, NQO1*, pri-miR-34a* and *pri-miR-34b/c* by salicylate was largely abolished after DOX-mediated activation of a conditional *c-MYC* allele in DLD-1/*pRTR-c-MYC-VSV* cells (Fig. 8G–I and Supplementary Fig. S8D, E). Conversely, suppression of *c-MYC* by specific siRNA pools resulted in elevated expression of NQO1 protein and *pri-miR-34a* (Fig. 8J, K). In summary, these results imply that the suppression of c-MYC is necessary for the activation of NRF2 and induction of *miR-34a* and *miR-34b/c* by salicylate.

## Discussion

Here we characterized the activation of an AMPK-NRF2-miR-34a/b/c axis by salicylate as a novel mechanism of salicylate-mediated suppression of CRC (see Fig. 8L). Our results suggest that this mechanism plays a central role in mediating the tumor suppressive effects of salicylate since the deletion or suppression of miR-34a/b/c prevented the salicylate-mediated suppression of CRC cell viability, migration, invasion, and metastasis. We recently showed that curcumin induces *miR-34a/b/c* expression via the activation of NRF2 [27]. NRF2 is a transcription factor that regulates the cellular response to stress [31]. Under normal conditions, NRF2 is rapidly degraded and sequestered in the cytoplasm by a KEAP1-CUL3-RBX1 complex [32]. In response to stress NRF2 is released from the complex and translocates to the nucleus, where it binds to ARE motifs and induces the expression of corresponding genes [33]. The activation of NRF2 by curcumin was mediated by the induction of reactive oxygen species (ROS). Here we found that salicylate also activates NRF2. However, salicylate achieves this by activating AMPK in the absence of any detectable generation of ROS. Since ROS may cause DNA damage, which may lead to cell death or induce mutations, not only in cancer cells, but also in normal cells, using salicylate as a chemo-preventive drug may have less side effects than curcumin.

AMPK is a sensor of cellular energy status and a regulator of energy balance [34]. AMPK is composed of a catalytic α subunit, as well as the regulatory β and γ subunits [35]. It has been shown that salicylate binds to AMPK between the α and β subunits [36], which causes a conformational change that promotes the phosphorylation of Thr172 by upstream kinases while inhibiting dephosphorylation by phosphatases [35]. The activation of AMPK by salicylate was abolished by the loss or mutation of the AMPK β1 subunit [37]. Our results show that both AMPK α1 and AMPK β1 subunits are important for the induction of *miR-34a/b/c* by salicylate, since the suppression of either subunit prevented the activation of NRF2 and the induction of *miR-34a/b/c*.

It has previously been shown that salicylate suppresses the expression of c-MYC [12]. However, the mechanism remained unknown. Here, the repression of c-MYC by salicylate was p53-independent. The inhibition of AMPK by siRNA or chemical inhibitors abolished the repression of c-MYC by salicylate, suggesting that salicylate suppresses the expression of c-MYC via AMPK. Interestingly, another inducer of AMPK, Metformin, also down-regulates c-MYC expression and inhibits CRC cell proliferation [38]. Moreover, the AMPK activator AICAR suppresses c-MYC via SIRT1 and GSK3β [39]. Finally, another AMPK activator, 4-O-Methly-ascochlorin, suppresses c-MYC activity via mTOR [40]. Therefore, the suppression of c-MYC by AMPK probably involves multiple mechanisms. In addition, salicylate suppresses WNT signaling at multiple levels, i.e. by inhibition of COX-2 and by activation of protein phosphatase 2A, which promotes β-catenin degradation [41]. Since c-*MYC* is a target gene of the WNT/TCF4 pathway [42], the suppression of c-*MYC* by salicylate might also be mediated by inhibition of WNT signaling.

Interestingly, a single nucleotide polymorphism (SNP) rs6983267 located on chromosome 8q24 that regulates the expression of c-MYC has been associated with the chemo-preventive effect of aspirin [43]. The protective effect of aspirin was confined to individuals with the T allele of the SNP, which was associated with reduced expression of c-MYC. The SNP affects a binding site for the WNT-regulated transcription factor TCF4, with the T allele showing weaker binding [44, 45]. The allele frequency of the T allele is high (49% among Europeans) and thus the majority of the population may benefit from the chemo-preventive use of aspirin [41].

We showed that the ectopic expression of c-MYC prevents the activation of NRF2 and the induction of *miR-34a/b/c* by salicylate. It has been shown that c-MYC interacts with NRF2, which prevents the binding of NRF2 to the ARE elements and the induction of NRF2 target genes [30, 46]. Therefore, the binding of c-MYC to NRF2 might sequester NRF2 from the *miR-34a/b/c* promoters and thereby prevents the induction of *miR-34a/b/c* expression by NRF2. We showed that salicylate on one hand activates NRF2 and on the other hand suppresses the expression of c-*MYC*. Our results suggest that both mechanisms contribute to the salicylate-mediated induction of *miR-34a/b/c*.

Aspirin was shown to be effective in cancer prevention when given for extended periods [47–50]. Dominick J et al. [51]. determined that the Cmax is 25–53 μg /ml (160–332 μM) after intake of 650 mg aspirin as tablets. Since we intended to analyze the acute effects of salicylate, the main metabolite of aspirin, on CRC cells and characterize the mediators of these effects in periods covering a few days at most, we chose to apply a higher concentration (5 mM) of salicylate based on our IC_50_ value determination. In addition, a concentration of 5 mM was used in previous studies, which examined the effect of salicylate on CRC cells [21–24]. Presumably, lower doses of aspirin given over extended periods for cancer prevention exert the same or similar effects, which we observed here for 5 mM salicylate. In the future, in vivo analyses to confirm this assumption are warranted.

We challenge the notion that aspirin prevents cancer through a single, dominant pathway (the inhibition of COX enzymes [10]) and propose an integrative multi-pathway model for its mode of action, with miR-34a and miR-34b/c representing important effectors. As of today, more than 200 experimentally validated miR-34a/b/c target mRNAs have been identified [52]. In the future it will be important to identify the miR-34 targets that mediate the tumor suppressive effects of aspirin.

## Materials and methods

### Cell culture and treatments

The CRC cell lines (HCT116, HCT116 *miR-34a*−/−, HCT116 *miR-34a/b/c*−/−, RKO, SW48, SW480, DLD-1, SW620-*Luc2*, DLD1-*pRTR-c-MYC-VSV*) and Human colon fibroblast (CCD18-co) were cultured in McCoy’s 5A medium (Invitrogen, Carlsbad, CA, USA) with 10% fetal bovine serum (FBS) (Invitrogen) containing 100 units/ml penicillin and 0.1 mg/ml streptomycin at 20% O_2_, 5% CO_2_, and 37 °C. *p53*-deficient and *p53*-proficient HCT116 and RKO cells were a gift from Bert Vogelstein (Johns Hopkins Medical School, Baltimore, Maryland, USA) [53]. The isogenic wt and *miR-34a-* or *miR-34a/b/c*-deficient HCT116 cells were generated in our lab using a CRISPR/CAS9 approach [26]. HCEC-1CT immortalized human colonic epithelial cells (Evercyte GmbH) were maintained in DMEM medium supplemented with 2% FBS, 1 × N2 supplement (containing insulin, apolipoprotein, and sodium selenite; Thermo Fisher Scientific), 20 ng/ml epidermal growth factor (EGF; AF-100-15, PeproTech, part of Thermo Fisher Scientific), 1 μg/ml hydrocortisone (Sigma, Merck) and 100 units/ml penicillin and 0.1 mg/ml streptomycin (Gibco, Thermo Fisher Scientific). Sodium salicylate (Lot # SLCC 7389, Sigma-Aldrich) was dissolved in distilled H_2_O to a 600 mM stock solution and applied at 5 mM working concentration. Dorsomorphin (Compound C, ab120843, Abcam) was dissolved in DMSO with a concentration of 10 mM. siGENOME Human *NRF2* SMARTPOOL (Horizon Discovery, USA, #M-003755-02-0010), Human *PRKAA1* siRNA SMARTPOOL (Horizon Discovery, USA, #M-005027-02-0010), Human *PRKAB1* siRNA SMARTPOOL (Horizon Discovery, USA, # M-007675-00-0010), control siRNA pool (Horizon Discovery, USA), miRNA antagonist (*hsa-miR-34a-5p* inhibitor, Invitrogen, MH11030), and corresponding negative controls were transfected at a concentration of 10 nM using HiPerfect transfection reagent (Qiagen, Hilden, Germany).

### MTT assay

Cells were seeded at a density of 3000 cells per well in 96-well plates and treated with salicylate at different concentrations for 48 h. Then, 10 μl of MTT solution was added to each well and incubated for 4 h. This assay is based on reducing MTT (3-(4,5-dimethylthiazol-2-yl)-2,5-diphenyltetrazolium bromide) by viable cells to form a purple formazan product. Cells were treated with DMSO to release the formazan and the absorbance was measured at 570 nm using a Varioscan system (Thermo Fisher).

### Assessment of proliferation by real-time impedance

Cell proliferation was analyzed using the xCELLigence system. The cells were seeded at a density of 3 × 10^3^ cells in 100 μL per well of E-Plates®. After 24 h, cells were exposed to 5 mM salicylate. The xCELLigence system was used to monitor the cells for an additional 116 h. The system automatically calculates a parameter called “Cell Index” based on the measured impedance. Real-time monitoring of electrical impedance, represented as the cell index, provided continuous information about cell behavior and growth, which was further confirmed by manual cell counting at the end of the experiment.

### Cell cycle analysis by flow cytometry

HCT116 cell lines were seeded into 6-well plates at the density of 2 × 10^5^ cells/ml. The supernatant with detached cells was transferred into 15 ml tubes. Cells were harvested by adding trypsin and neutralized with 1 ml of fresh medium. The cell suspension was transferred to a 15 ml tube, centrifuged at 1200 rpm for 4 min, and the supernatant was discarded. The cells were washed with 3 ml of 1% BSA in PBS. 100 μl of Click-iT® fixative was added. The cells were incubated for 15 min at room temperature, protected from light. Next, cells were washed with 3 ml of 1% BSA in PBS and pelleted to remove the supernatant. The cells were resuspended in 100 μl of 1X Click-iT® saponin-based permeabilization solution and mixed well. Cells were incubated for 15 min, followed by the addition of 0.5 ml of Click-iT® reaction cocktail to each tube and incubated for 30 min at room temperature, protected from light. Cells were resuspended in Propidium Iodide (PI) staining solution and analyzed by flow cytometry using a CFlow6 device (Accuri, Ann Arbor, MI).

### RNA isolation and quantitative real-time polymerase chain reaction (qPCR)

Total cellular RNA was isolated and purified from CRC cell lines according to the manufacturer’s instructions (High Pure RNA Isolation Kit; Roche). 1 µg of RNA was reverse transcribed using a Verso cDNA Synthesis Kit from Thermo Fisher Scientific (Waltham, MA, USA). qPCR was conducted using Fast SYBR Green Master Mix from Applied Biosystems on a LightCycler 480 system from Roche. The data obtained from qPCR analysis was normalized to the expression of the housekeeping genes *GAPDH* or *β-actin*. The analysis was performed using the ΔΔCt method. The primer sequences used in the qPCR assay are provided in Supplementary Table S1.

### Detection of apoptosis by flow cytometry

HCT116 cells were seeded into 6 well plates at a cell density of 2 × 10^5^ cells per well. After 24 h cells were treated with 5 mM of salicylate for indicated times. Cells were harvested, washed with HBSS, and resuspended in 1 X binding buffer. FITC Annexin V and PI were added and incubated for 15 min at room temperature in the dark. After addition of 1x binding buffer, cells were analyzed by flow cytometry using a CFlow6 device (Accuri, Ann Arbor, MI).

### Immunofluorescence and confocal laser-scanning microscopy

The cells were first cultured on glass cover slides and then fixed in 4% paraformaldehyde/PBS for 15 min, permeabilized with 0.2% Triton X-100 for 5 min and blocked in 1% BSA/PBS for 1 h at room temperature. Next, the cells were incubated with a primary antibody against NRF2 (D1Z9C, #12721, Cell Signaling Technology) for 1 h at room temperature, washed three times with PBS-Tween, and then incubated with a Cy3-labeled secondary antibody (ab6939, Abcam). The chromatin was stained with DAPI (Roth). Finally, confocal laser scanning microscopy (CLSM) was used to acquire images of the cells. For imaging a LSM700 microscope with a Plan Apochromat 20×/0.8 M27 objective and ZEN 2009 software (Zeiss) was used.

### Western blot analysis

Cells were washed by HBSS and lysed in RIPA lysis buffer (50 mM Tris/HCl, pH 8.0, 250 mM NaCl, 1% NP40, 0.5% [w/v] sodium deoxycholate, 0.1% SDS, complete mini protease inhibitors [Roche, Basel, Switzerland], PhosSTOP Phosphatase Inhibitor Cocktail Tablets [Roche]). Lysates were sonicated for 5 s and centrifuged at 13,000 rpm for 20 min at 4 °C. Supernatants containing proteins were transferred to new tubes and quantified by Pierce™ BCA Protein Assay Kit (Termo Fisher Scientifc). 40 μg of protein from each sample was loaded and separated on 10% SDS-acrylamide gels. Gel electrophoresis and transfer to Immobilon PVDF membranes (Millipore, Burlington, MA, USA) were performed according to manufacturer’s instructions (BioRad Laboratories, Hercules, CA). Membranes were blocked with non-fat dry milk. For immuno-detection, the membrane was incubated with the primary antibodies listed in Supplementary Table S3. The signal from the HRP-conjugated secondary antibody was generated by enhanced chemi-luminescence (Millipore) and detected using a LI-COR Odyssey Fc imaging system (LI-COR, Lincoln, NE). Antibodies used in the Western blot are provided in Supplementary Table S3.

### Wound healing assay

A wound healing assay was used to assess migration of cells. 70 μl cell suspension at a density of 1 × 10^6^ cells/ml was seeded into a 2-well culture-Insert (80241; IBIDI, Martinsried, Germany). After attaching, mitomycin C (10 ng/ml) was added 2 h before removal of the insert. After 0 and 24 h the scratched area was documented using an Axiovert Observer Z.1 microscope with an AxioCam MRm camera and Axiovision software (Axiovs 40 Version 4.8.0.0, Zeiss, Oberhochen, Germany). The cell-free area was determined using ImageJ software.

### Modified Boyden-chamber assay

For determination of invasion, 60 µl of matrigel in serum-free medium (BD Biosciences, East Rutherford, NJ) at a concentration of 300 µg/ml was added to the chamber 4 h before adding cells. Then 5 × 10^4^ cells were seeded in the upper chamber (membranes with 8.0 µm pore size, Corning, NY). Salicylate was added to the top chamber at a final concentration of 5 mM. Then 500 µl of 10% FBS medium was added to the lower chamber. After 48 h the invaded cells located on the lower surface of the cell culture inserts were fixed, stained with crystal violet, and counted. Fold changes in invasive cells were calculated by normalizing them to the corresponding control group.

### Chromatin immunoprecipitation

Chromatin immunoprecipitation (ChIP) analysis was performed using HCT116 *p53*−/− cells. The cells were treated with salicylate for 48 h before cross-linking. ChIP experiments were performed using the iDeal ChIP-qPCR kit from Diagenode, Belgium, following the manufacturer’s instructions. Sequences of qChIP primers and antibodies are provided in Supplementary Tables S2 and S3.

### Metastases formation in a xenograft mouse model

SW620 cells stably expressing *Luc2* were described previously [54]. After the treatments Luciferase-tagged cells were washed with HBSS for 3 times and the number of living cells was determined after staining with trypan blue. 4 ×10^6^ living cells were injected into the lateral tail vein of NOD/SCID mice using 25-gauge needles. Starting from the second week after injection, the mice were injected intraperitoneally (i.p.) with D-luciferin at a dose of 150 mg/kg. The mice were then subjected to weekly bioluminescent imaging using the IVIS Illumina System from Caliper Life Sciences. The imaging was performed for 5 min. Five weeks after the initial tail vein injection, the mice were sacrificed, and their lung tissues were examined for the presence of metastases using hematoxylin and eosin (H&E) staining. All animal experiments and analyses were conducted in compliance with the regulations and guidelines set by the Government of Upper Bavaria, Germany (Approval number 55.2-2532.vet_02-18-57).

### DCFDA/H2DCFDA - cellular ROS assay

Reactive oxygen species (ROS) were analyzed using DCFDA/H2DCFDA staining (Cellular ROS Assay Kit (ab113851, Abcam). HCT116 cells were seeded in 96-well plates at a density of 10^4^ cells per well. 24 h after seeding, cells were treated with or without salicylate for 48 h. As a positive control, tBHP (tert-Butyl hydroperoxide) was added to the HCT116 cells for 4 h before staining. After the indicated treatment period, the medium was removed, and 100 µl of 1X buffer was added to each well. Subsequently, the cells were stained with DCFDA solution (100 µl/well) and incubated for 45 min at 37 °C in the dark. After the staining period, the DCFDA solution was removed, and the plates were immediately analyzed using a fluorescence plate reader with excitation/emission wavelengths set at 485/535 nm, which corresponds to the excitation and emission wavelengths of the DCFDA dye. To analyze the images obtained from the plate reader Image J software was used.

### Statistical analysis

The GraphPad Prism software was used for statistical analyses. The statistical differences between two groups were calculated using a Student’s *t* test (two-tailed; unpaired). When more than 2 groups were compared, a one-way analysis of variance (ANOVA) combined with a Tukey multiple comparison post-test was used. Asterisks generally indicate: *p < 0.05, **p < 0.01 and ***p < 0.001, n.s. not significant.

### Supplementary information

reproducibility checklist

Original Data File

Supplemental Material

## Data Availability

All data, analytic methods, and study materials will be made available to other researchers upon reasonable request.
