# Supplementary material for: Salicylate induces AMPK and inhibits c-MYC to activate a NRF2/ARE/miR-34a/b/c cascade resulting in suppression of colorectal cancer metastasis
Source: Cell Death Dis. 2023 Oct 28;14(10):707. doi: 10.1038/s41419-023-06226-9 (PMC10613307; doi:10.1038/s41419-023-06226-9)

Original Western blots/Uncropped membranes

Uncropped membranes for Figure 1F

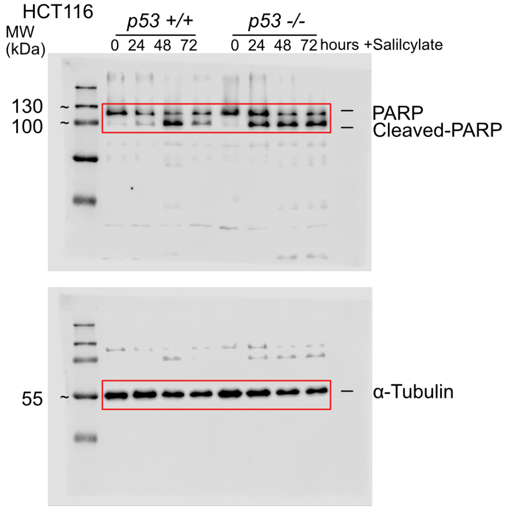

Uncropped membranes for Figure 2H

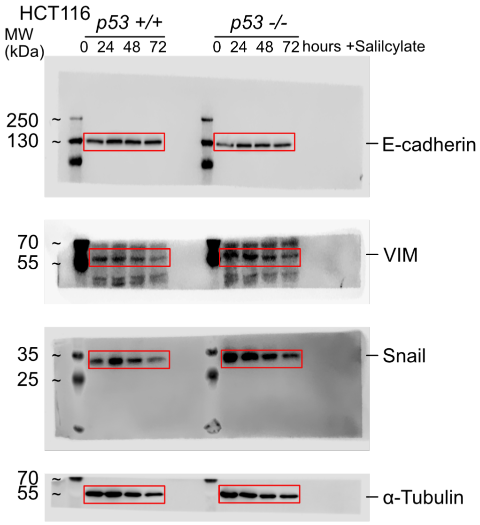

Uncropped membranes for Figure 4H

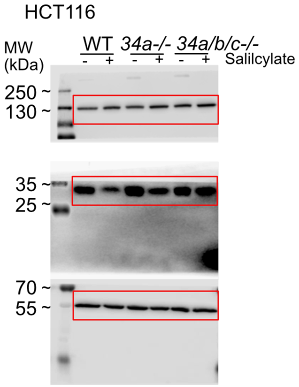

Uncropped membranes for Figure 6C

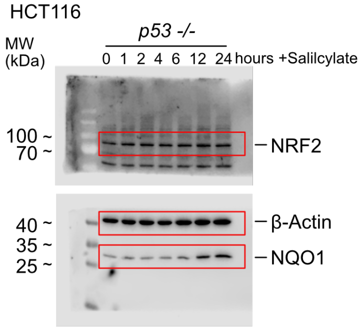

Uncropped membranes for Figure 6E

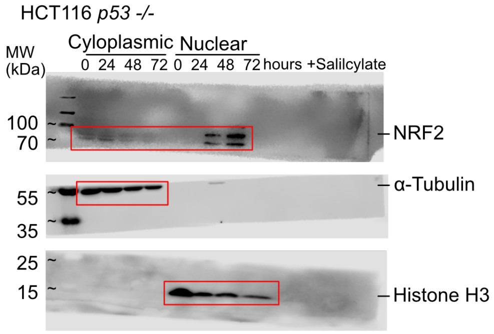

Uncropped membranes for Figure 6J

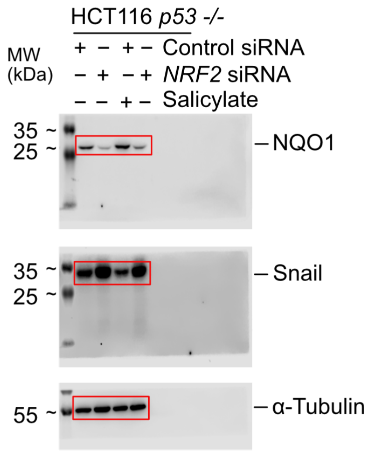

Uncropped membranes for Figure 7A

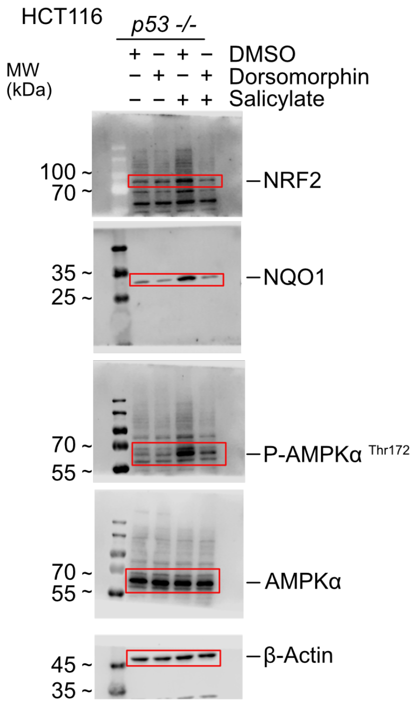

Uncropped membranes for Figure 7C

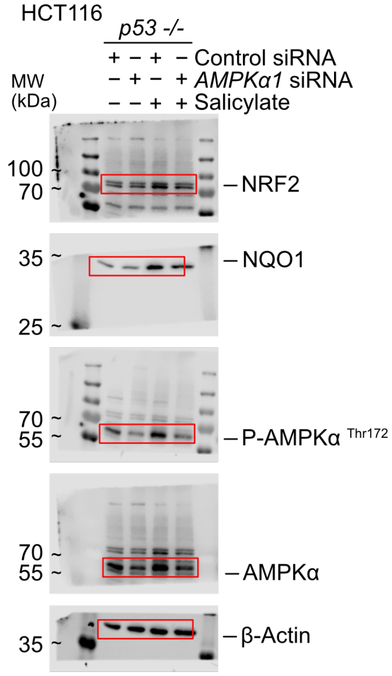

Uncropped membranes for Figure 7F

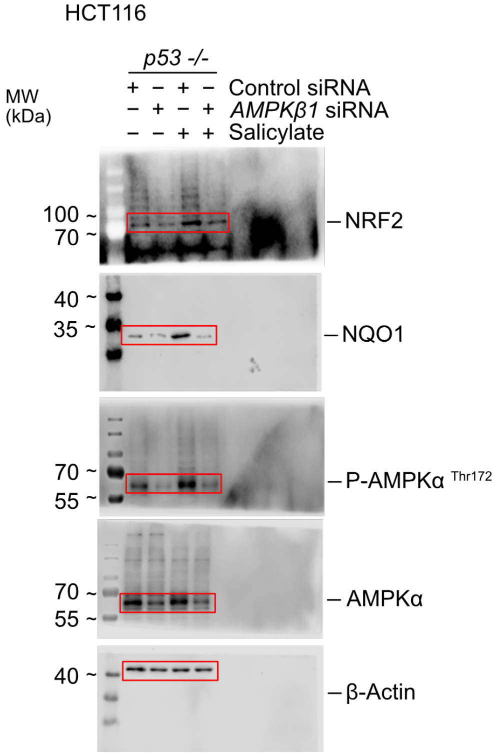

Uncropped membranes for Figure 8C

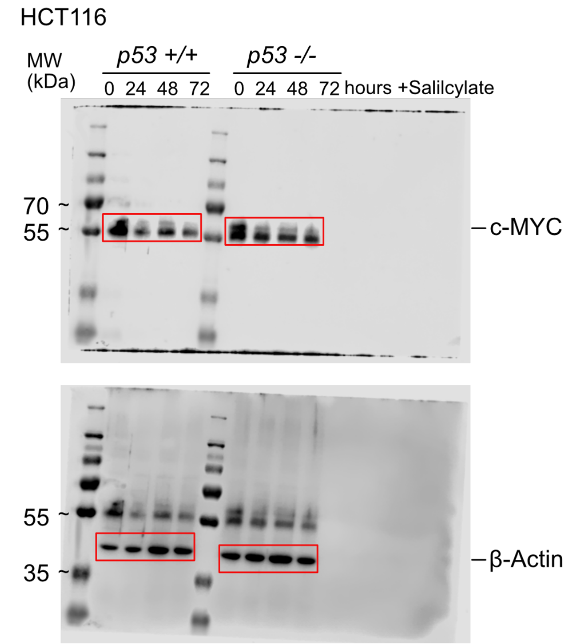

Uncropped membranes for Figure 8D

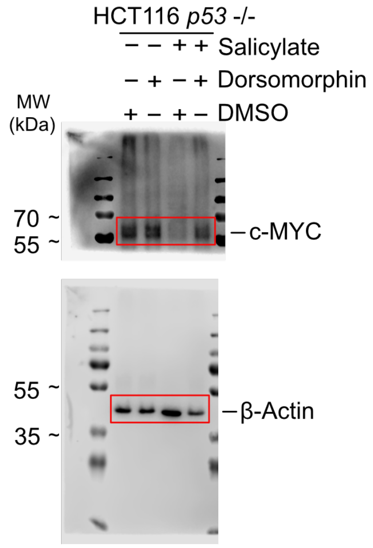

Uncropped membranes for Figure 8E

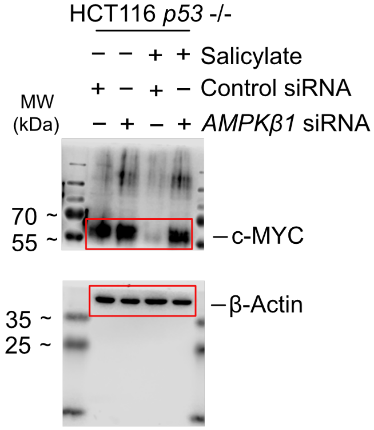

Uncropped membranes for Figure 8F

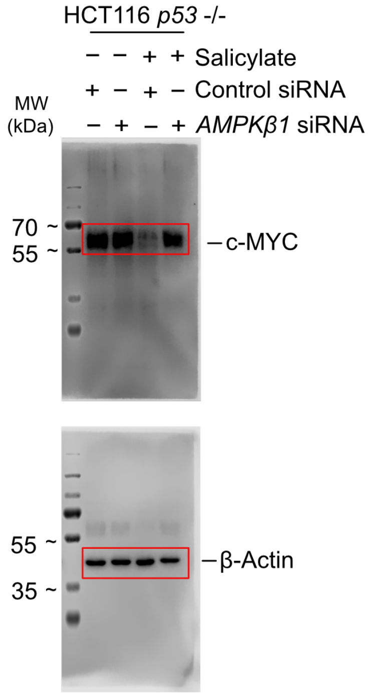

Uncropped membranes for Figure 8G

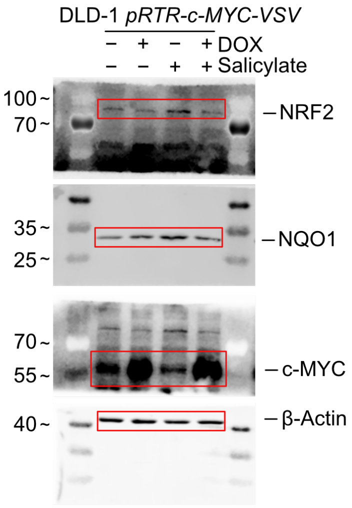

Uncropped membranes for Figure 8I

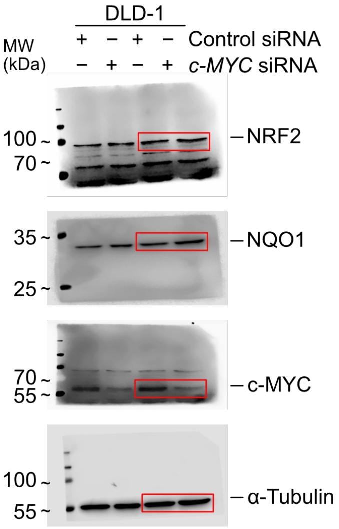

Uncropped membranes for Figure S1C

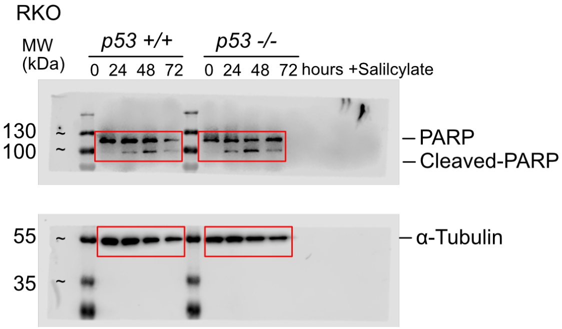

Uncropped membranes for Figure S2E

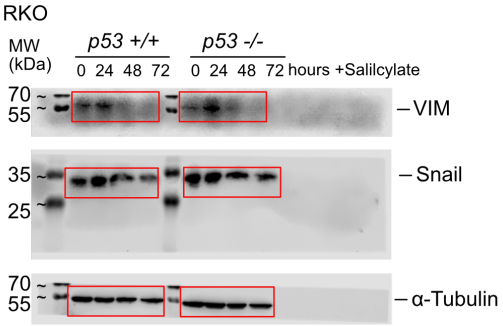

Uncropped membranes for Figure S7A

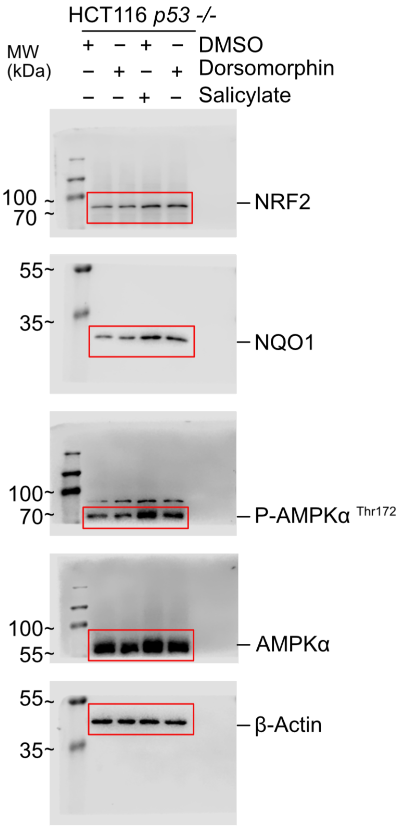

Uncropped membranes for Figure S7B

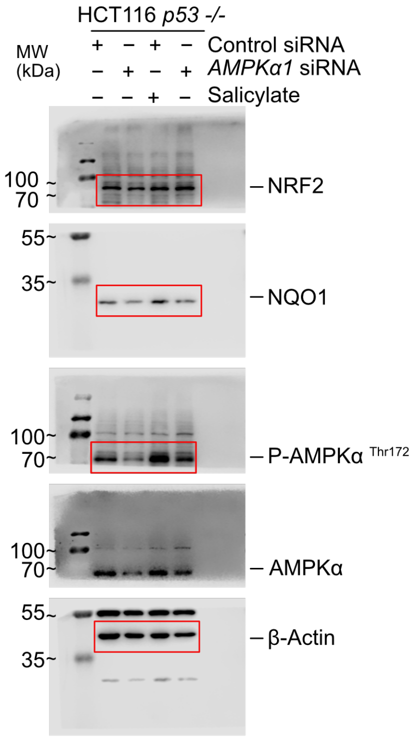

Uncropped membranes for Figure S7E

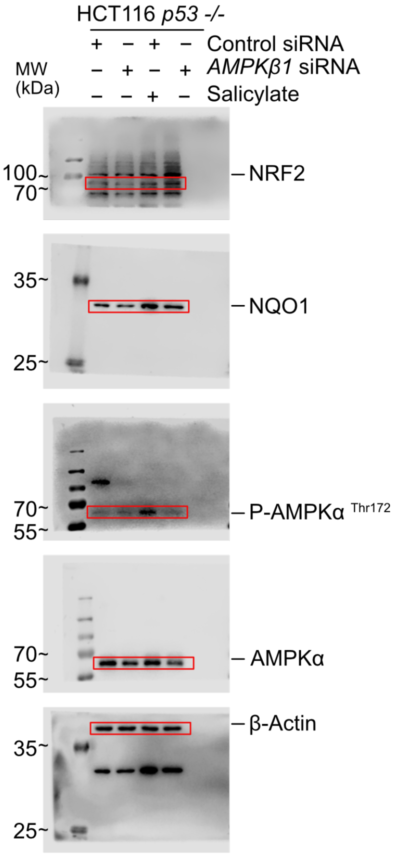

Uncropped membranes for Figure S8A

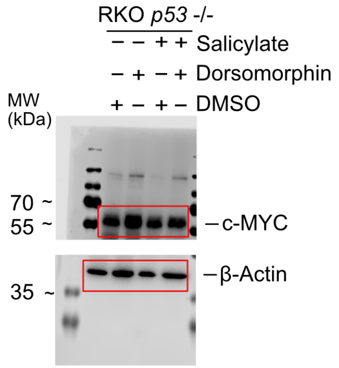

Uncropped membranes for Figure S8B

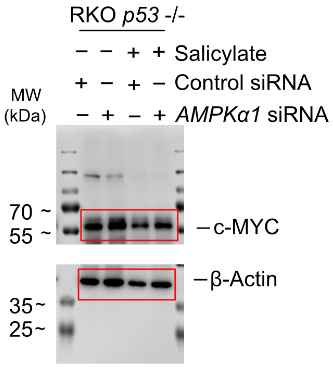

Uncropped membranes for Figure S8C

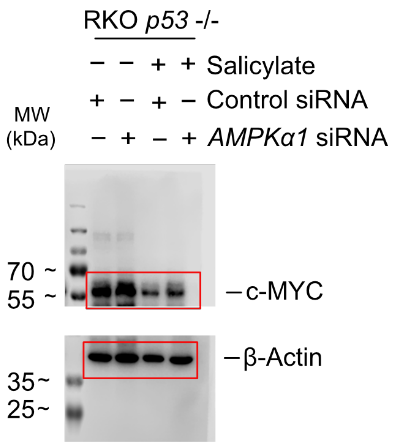

Supplement: Supplementary file 2 — Original Data File [file 41419_2023_6226_MOESM2_ESM.pdf]
