# Supplementary material for: Salicylate induces AMPK and inhibits c-MYC to activate a NRF2/ARE/miR-34a/b/c cascade resulting in suppression of colorectal cancer metastasis
Source: Cell Death Dis. 2023 Oct 28;14(10):707. doi: 10.1038/s41419-023-06226-9 (PMC10613307; doi:10.1038/s41419-023-06226-9)
Supplement: Supplementary file 3 — Supplemental Material [file 41419_2023_6226_MOESM3_ESM.pdf]

Supplementary material

Salicylate induces AMPK and inhibits c-*MYC* to activate a NRF2/ARE/*miR-34a/b/c* cascade resulting in suppression of colorectal cancer metastasis

Chunfeng Liu, Matjaz Rokavec, Zekai Huang and Heiko Hermeking

Inventory of Supplementary Information:

- **Supplementary Figure 1**
  Related to Figure 1
  Salicylate inhibits cell viability and induces apoptosis in CRC cells

- **Supplementary Figure 2**
  Related to Figure 2
  Effects of salicylate on migration, invasion and MET in CRC cells

- **Supplementary Figure 3**
  Related to Figure 3
  Salicylate up-regulates *pri-miR-34a*/*b/c* and mature miR-34a independent of p53

- **Supplementary Figure 4**
  Related to Figure 4
  miR-34a and miR-34-b/c mediate the effects of salicylate in CRC cells

- **Supplementary Figure 5**
  Related to Figure 5
  Salicylate inhibits migration and invasion of SW620-*Luc2* cells in a miR-34a-dependent manner

- **Supplementary Figure 6**
  Related to Figure 6
  Salicylate inhibits migration and invasion in a NRF2-dependent manner

- **Supplementary Figure 7**
  Related to Figure 7
  Salicylate activates NRF2 via AMPK in CRC cells

- **Supplementary Figure 8**
  Related to Figure 8
  Suppression of c-MYC by salicylate is required for NRF2-mediated induction of *miR-34a/b/c*

- **Supplementary Table 1**
  Oligonucleotides used for qPCR

- **Supplementary Table 2**
  Oligonucleotides used for qChIP

- **Supplementary Table 3**
  List of antibodies

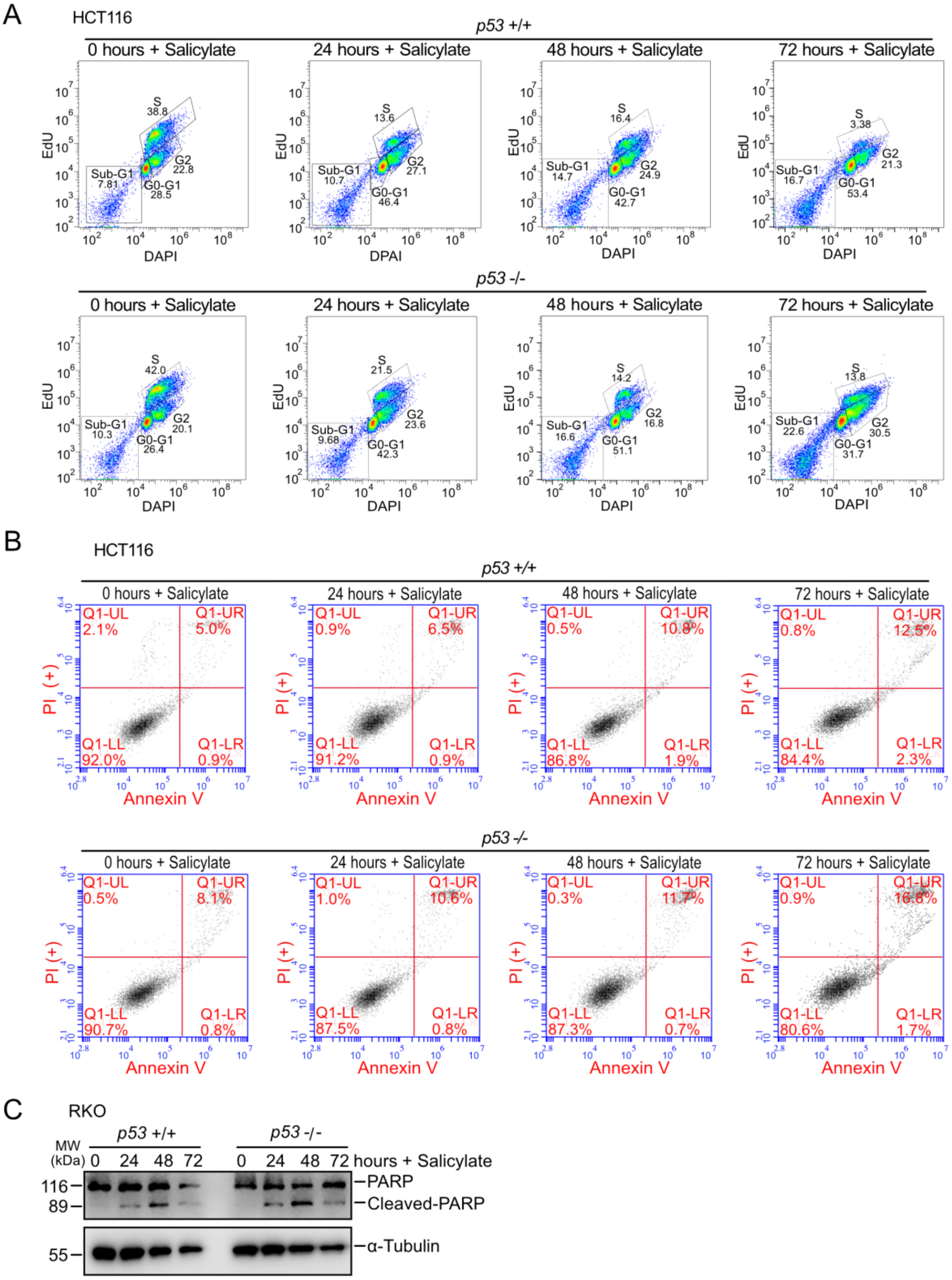

Figure S1: Salicylate inhibits cell viability and induces apoptosis in CRC cells
A. Cell cycle analysis by flow cytometry after staining of HCT116 cells with EdU. B. HCT116 cells were treated with 5 mM salicylate at the indicated time points and apoptotic cells were detected by Annexin V-FITC/PI staining. C. Detection of cleaved PARP/PARP protein after treatment salicylate at the indicated time-points in RKO cells by Western blot analysis.

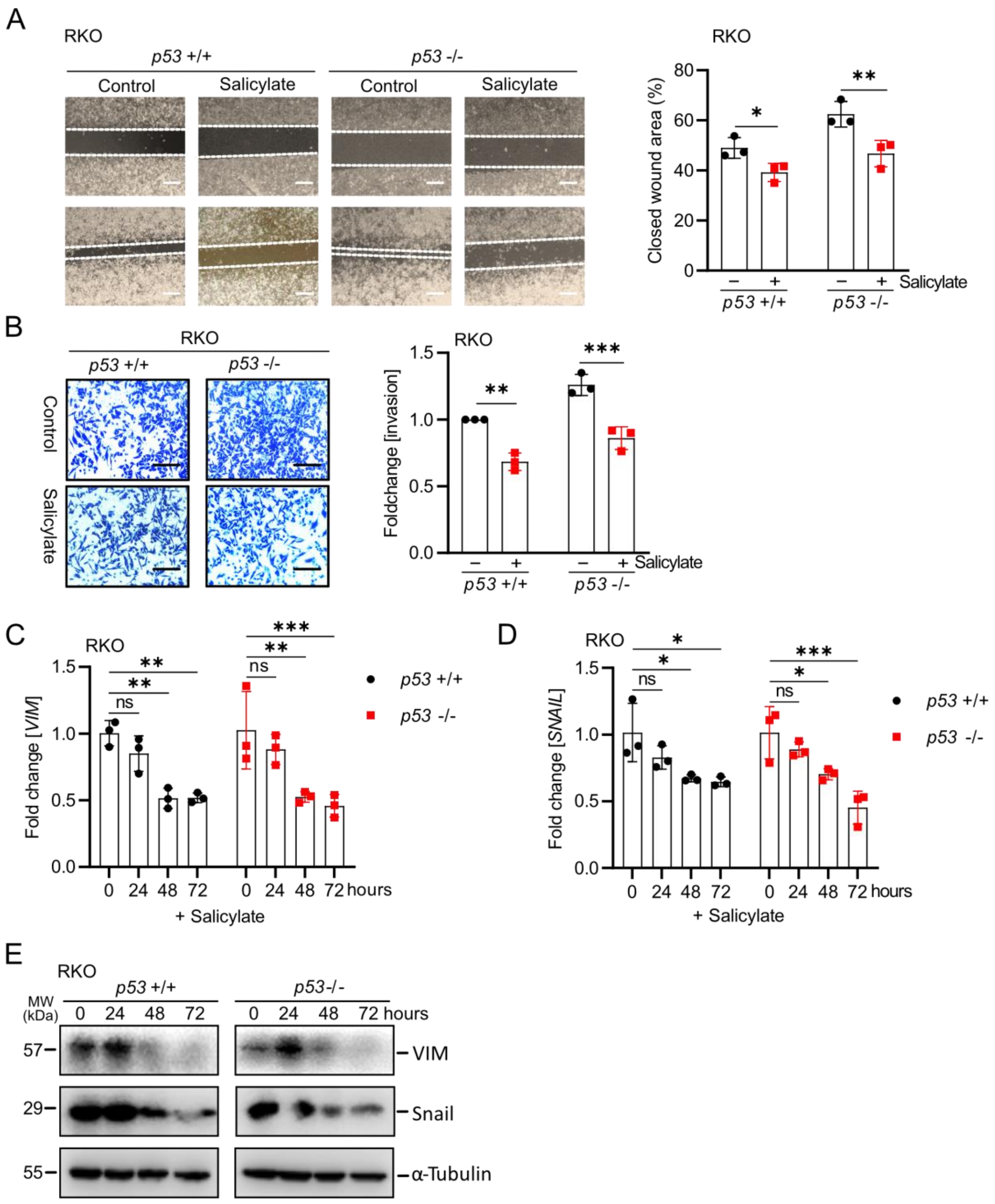

Figure S2: Effects of salicylate on migration, invasion and MET in CRC cells

A. Wound healing assay: the scratch width was determined 24 hours after the indicated treatment of RKO cells (left panel). Results represent the mean (%) of wound closure (right panel). Scale bars: 200 µm. B. Invasion was determined in a modified Boyden-chamber assay 48 hours after the indicated treatments of RKO cells (left panel). Fold changes in invasion were calculated by normalizing them to the corresponding control group (right panel). Scale bars: 100 µm. C-E. Analysis of mRNA and protein levels of MET markers 48 hours after the indicated treatments. In panels (A-D) the mean ± SD (n=3) is provided. * $P < 0.05$, ** $P < 0.01$, *** $P < 0.001$, and ns = not significant.

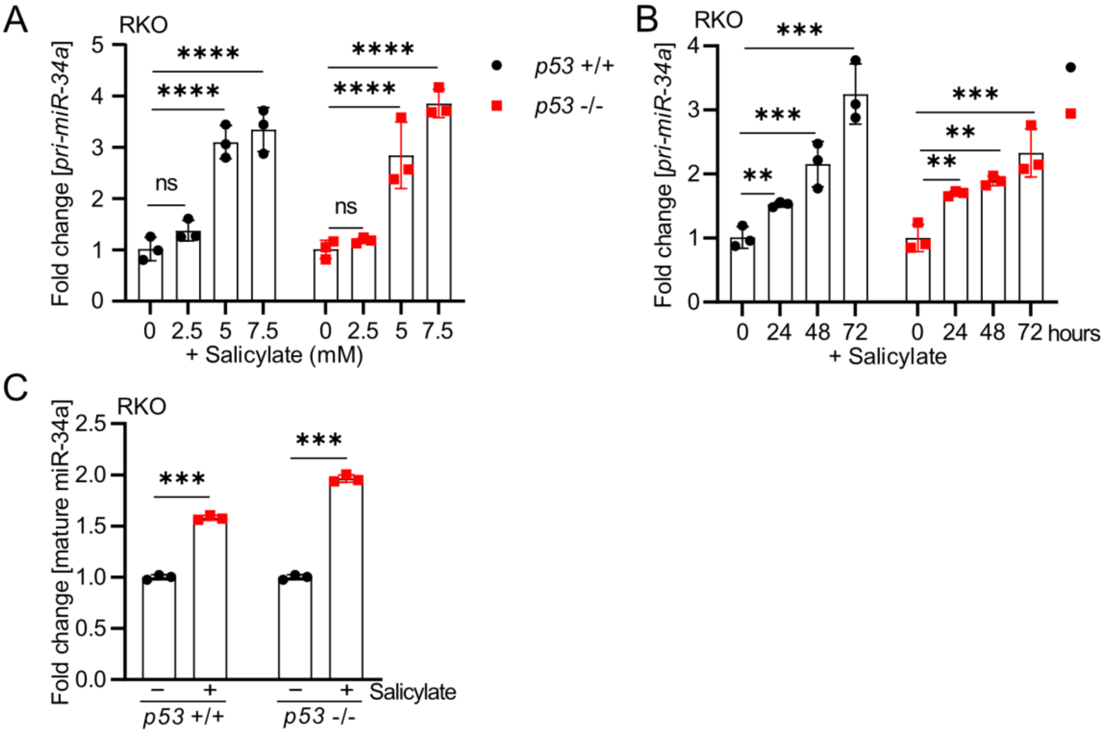

Figure S3: Salicylate up-regulates *pri-miR-34a/b/c* and mature miR-34a independent of p53

A-C. The expression of *pri-miR-34a* (A), *pri-miR-34b/c* (B), and mature miR-34a (C) after treatment of the indicated cells with salicylate for the indicated periods. In panel (A-C) the mean ± SD (n=3) is provided. ** P < 0.01, *** P < 0.001, *** P < 0.0001, ns = not significant.

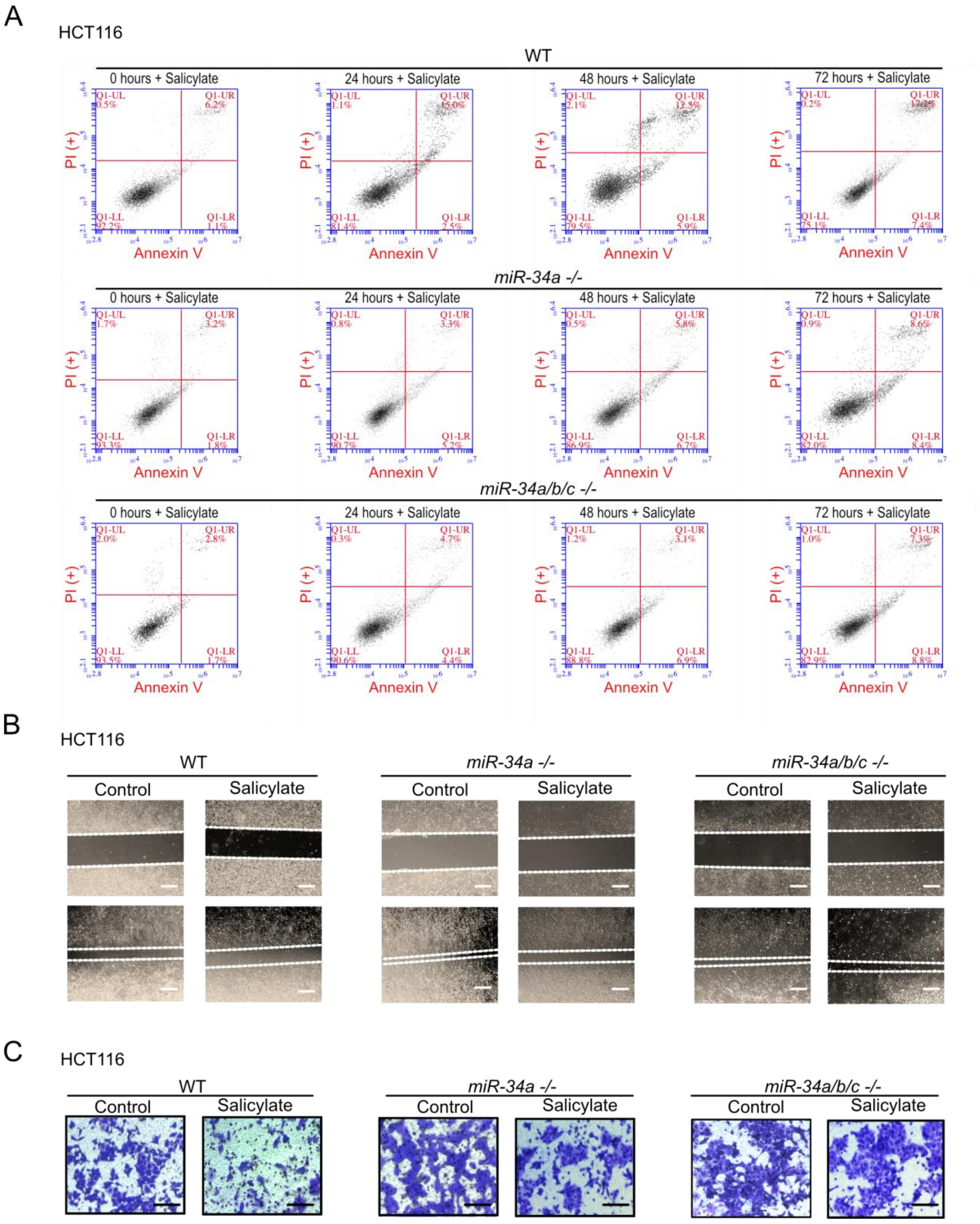

Figure S4: *miR-34a* and *miR-34b/c* mediate the effects of salicylate in CRC cells
A. Flow cytometric detection of Annexin V-FITC and PI staining after treatment of indicated cells with salicylate for indicated time points. B. Wound healing assay of indicated cells after treatment with salicylate for 24 hours. Scale bars: 200 µm. C. Modified Boyden-chamber assay for determination of invasion of indicated cells after treatment with salicylate for 48 hours. Scale bars: 100 µm.

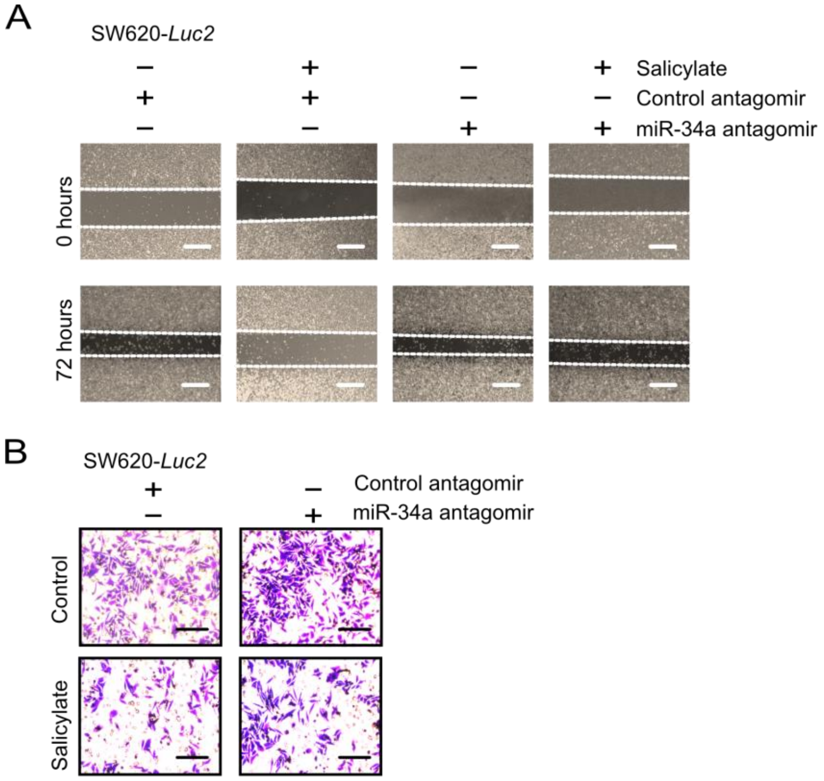

Figure S5: Salicylate inhibits migration and invasion of SW620-*Luc2* cells in a miR-34a-dependent manner

A. Wound healing assay of indicated cells after treatment with salicylate for 72 hours. Scale bars: 200 µm. B. Invasion was determined in a modified Boyden-chamber assay. The indicated cells were treated with salicylate for 48 hours. Scale bars: 100 µm.

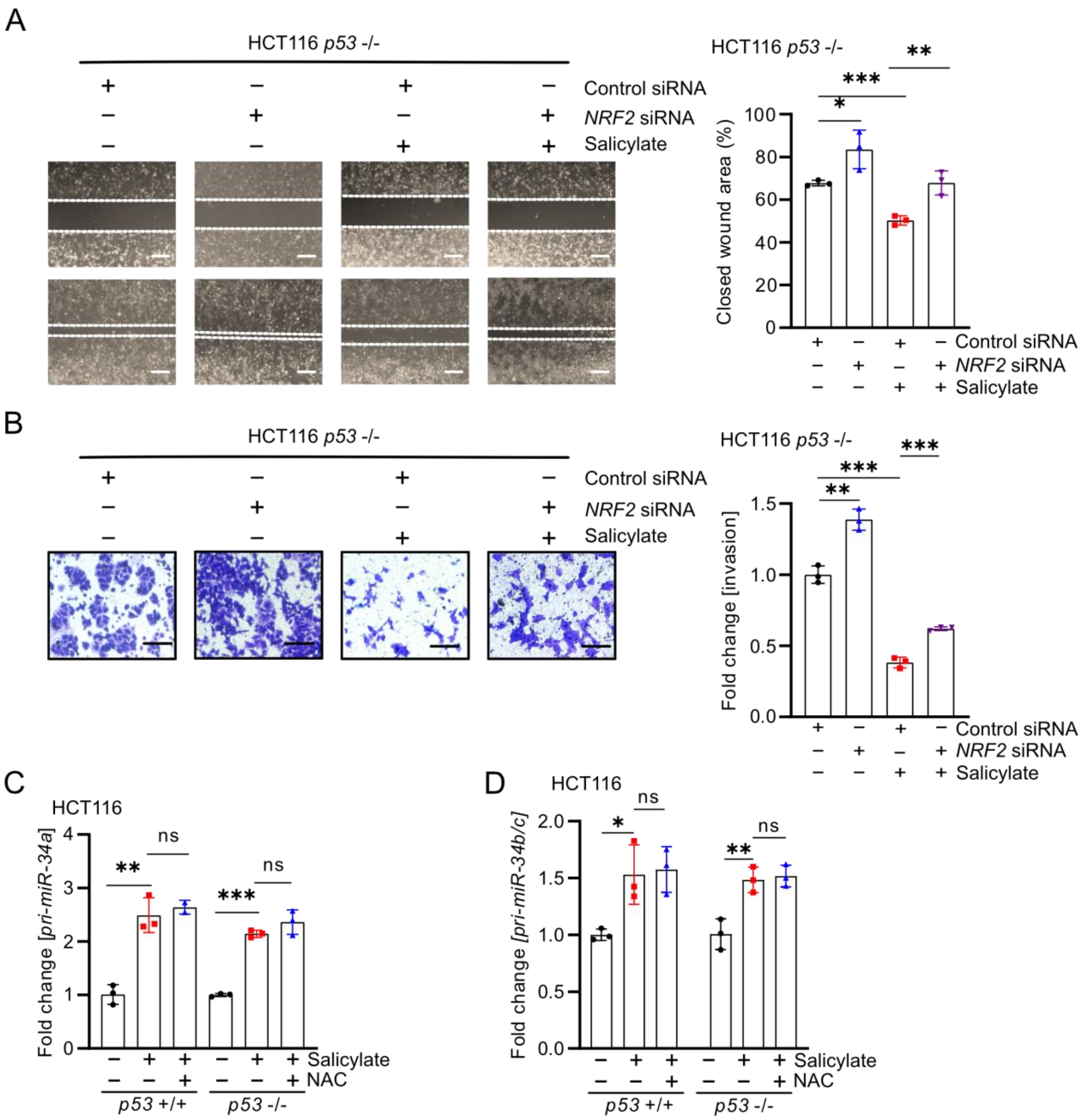

Figure S6: Salicylate inhibits migration and invasion in a NRF2-dependent manner

A. Wound healing assay of HCT116 *p53* -/- cells 24 hours after the indicated treatments (left panel). Scale bars: 200 µm. Results represent the mean (%) of wound closure (right panel). B. Determination of invasion in a modified Boyden-chamber assay of HCT116 *p53* -/- cells 48 hours after the indicated treatments. Scale bars: 100 µm. Fold changes in invasive cells were calculated by normalizing them to the corresponding control group (right panel). C-D. qPCR analysis of *pri-miR-34a* (C) and *pri-miR-34b/c* (D) in HCT116 cells for 48 hours after treatment with salicylate and/or NAC. In panels A-D the mean ± SD (n=3) is provided. * $P < 0.05$, ** $P < 0.01$, and *** $P < 0.001$.

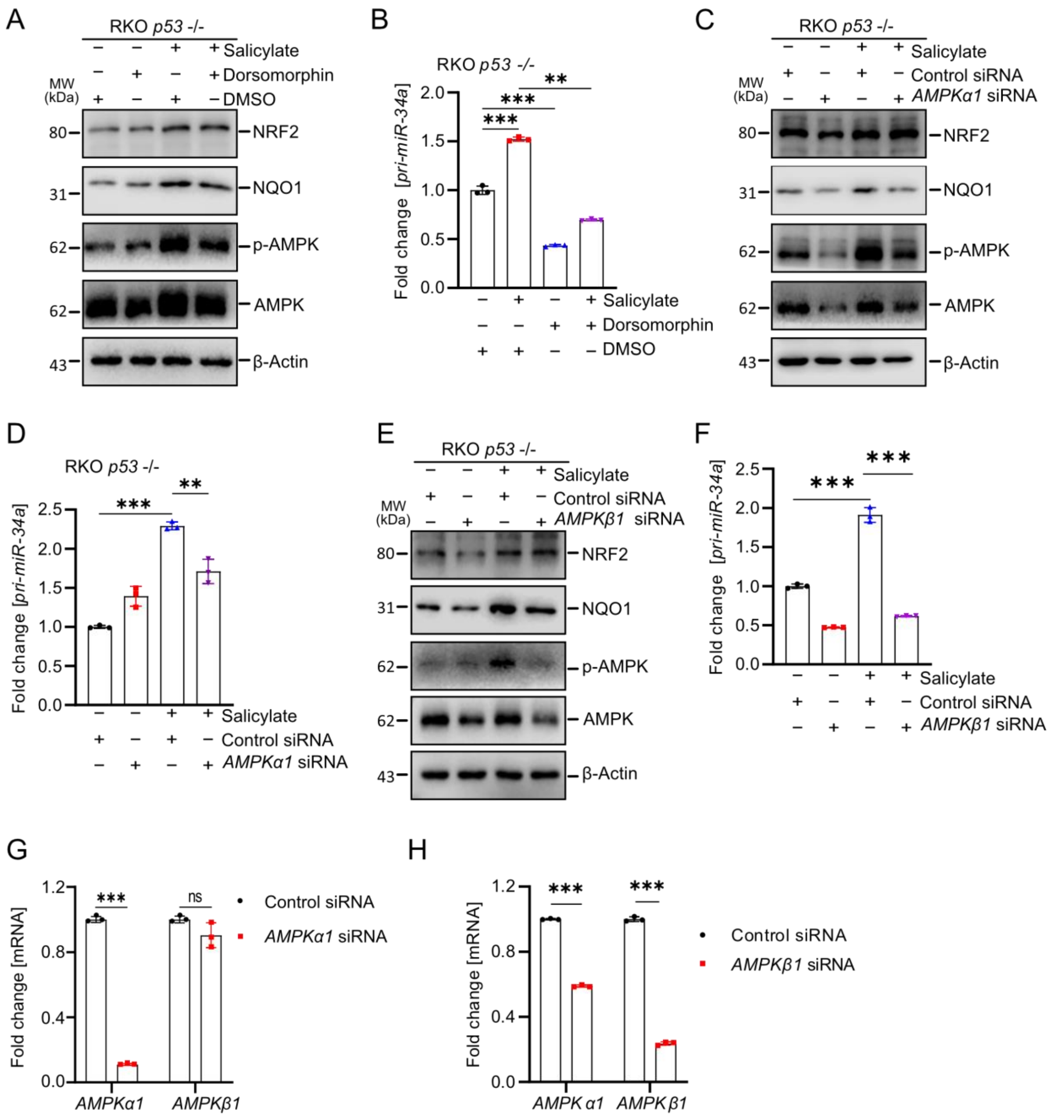

Figure S7: Salicylate activates NRF2 via AMPK in CRC cells

A. Detection of the indicated proteins by Western blot analysis 48 hours after the indicated treatments of RKO *p53 -/-* cells. B. qPCR analysis of *pri-miR-34a* 24 hours after the indicated treatments. C. Western blot analysis of the indicated proteins 24 hours after the indicated treatments. D. qPCR analysis of *pri-miR-34a* 24 hours after the indicated treatments. E. Western blot analysis of the indicated proteins 24 hours after the indicated treatment with siRNA pools. F. qPCR analysis of *pri-miR-34a* 24 hours after the indicated treatments. G and H. qPCR analysis of *AMPKα1* and *AMPKβ1* 48 hours after the indicated transfection with siRNA pools. In panels B, D, F, G, and H mean values ± SD (n=3) are provided. ** $P < 0.01$, *** $P < 0.001$, and ns = not significant.

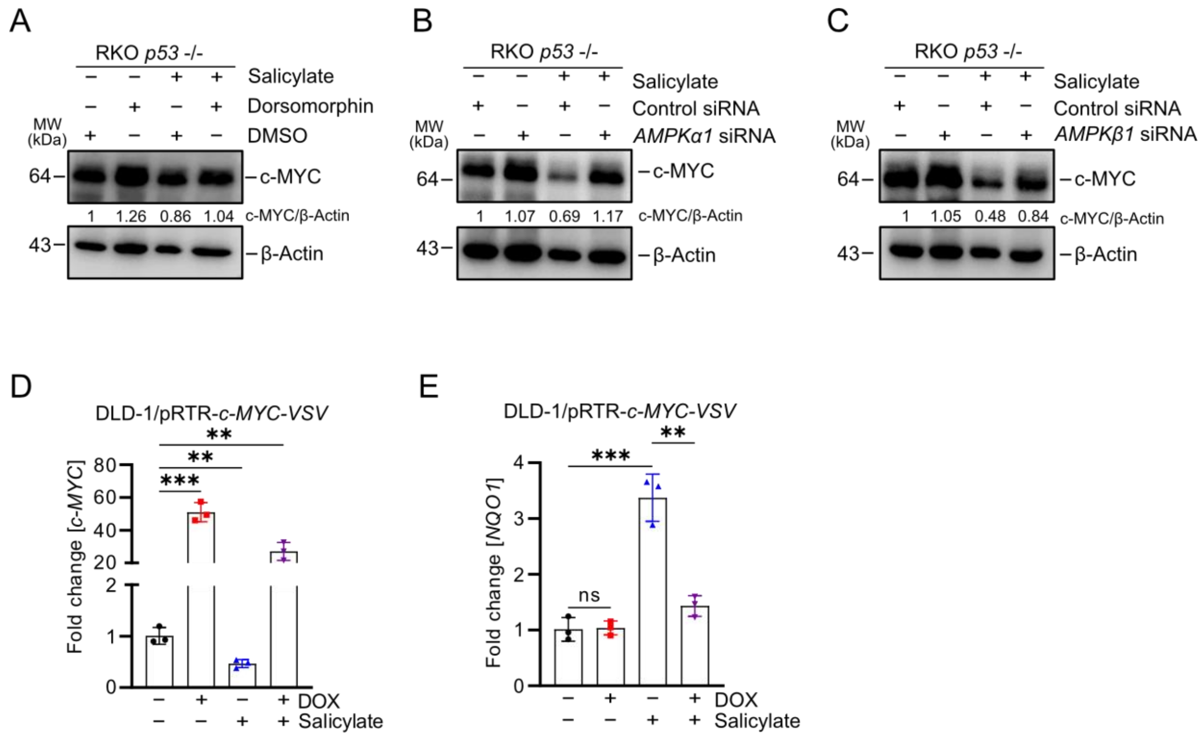

Figure S8: Suppression of c-MYC by salicylate is required for NRF2-mediated induction of *miR-34a/b/c*

A. Detection of the indicated proteins by Western blot analysis after the indicated treatments of RKO *p53 -/-* cells. B. Western blot analysis of the indicated proteins after the indicated treatments/transfections with siRNA pools. C. Western blot analysis of the indicated proteins after the indicated treatments/transfections with siRNA pools. D-E. qPCR analyses of *c-MYC* (D) and *NQO1* (E) expression in DLD-1 *pRTR-c-MYC-VSV* cells treated with salicylate or/and DOX (c-MYC on) for 48 hours.

A.

Supplemental Tables

Supplemental Table S1. Oligonucleotides used for qPCR

gene	forward (5' – 3')	reverse (5' – 3')
GAPDH	TGTTGCCATCAATGACCCCTT	CTCCACGACGTACTCAGCG
ACTIN	TGACATTAAGGAGAAGCTGTGCTAC	GAGTTGAAGGTAGTTTCGTGGATG
NQO1	TGGTCCCGTTTTGGCTATTCT	GAGACATGCTCCGTGGAGAC
NRF2	GCAAATGAGGTTTCTTCGGC	GGTCTTCTGTGGAGAGGATG
Pri-miR34a	CGTCACCTCTTAGGCTTGGA	CATTGGTGTCGTTGTGCTCT
Pri-miR34bc	GCTCGGTTTGTAGGCAGTGC	GATGGCAGTGGAGTTAGTGA
CDH1	CCCGGGACAACGTTTATTAC	GCTGGCTCAAGTCAAAGTCC
VIM	TACAGGAAGCTGCTGGAAGG	ACCAGAGGGAGTGAATCCAG
SLUG	GGGGAGAAGCCTTTTTCTTG	TCCTCATGTTTGTGCAGGAG
SNAIL	GCACATCCGAAGCCACAC	GGAGAAGGTCCGAGCACAC
AMPKα*1*	GGA GCC TTG ATG TGG TAG GA	TTT CAT CCA GCC TTC CAT TC
AMPKβ*1*	AAA AGT GCT CCG ACG TGT CT	ATG CCC GTG TCC TTG TTT AG
mature miR-34a	MS00003318 (QIAGEN)	
mature miR-34b	MS00031780 (QIAGEN)	
mature miR-34c	MS00003332 (QIAGEN)	

Supplemental Table S2. Oligonucleotides used for qChIP

gene	forward (5' – 3')	reverse (5' – 3')
MiR-34a (A)	GGACTCCCGCAAAATCTCCA	CACGAGCAGGAAGGAGGAC
MiR-34a (B)	TTACCCCTGGGACCGAGAGA	AGAATCTGTTGCGATGAAATCACT
MiR-34a (C)	TGTCTCAGAACGAGACAGTGG	CCGACTTCGTCCTCTTAGTGA
MiR-34b/c (D)	TGTTGTCTCCAATTGTCTCCA	AGATCGTGCCACTGCACTC
NQO1	ATTCGTCTCCACGGAGCAT	CATGCCCTTTTAGCCTTGG
16q22	CTACTCACTTATCCATCCAGGCTAC	ATTTCACACACTCAGACATCACAG

Supplemental Table S3. List of antibodies

Primary antibodies

epitope	catalog no.	company	use	dilution	source
PARP	# 9532	Cell Signaling Technology	WB	1:1000	rabbit
NRF2	# ab92946	Abcam	WB	1:1000	rabbit
α-Tubulin	# T-9026	Sigma-Aldrich	WB	1:1000	mouse
β-Actin	# 4967	Cell Signaling Technology	WB	1:1000	rabbit
H3	# 9715	Cell Signaling Technology	WB	1:1000	rabbit
NQO1	# sc-32793	Santa Cruz	WB	1:1000	mouse
NRF2	# 12721	Cell Signaling Technology	ChIP<br>IF	1:200<br>1:400	rabbit
p- AMPKα (Thr172)	# 2535	Cell Signaling Technology	WB	1:1000	rabbit
AMPKα	#5831	Cell Signaling Technology	WB	1:1000	rabbit
E-cadherin	# sc-8426	Santa Cruz	WB	1:1000	mouse
Vimentin (VIM)	#3932	Cell Signaling Technology	WB	1:500	rabbit
Snail	#3879	Cell Signaling Technology	WB	1:500	rabbit
c-MYC	#sc-40	Santa Cruz	WB	1:1000	mouse
Rabbit IgG		Sigma-Aldrich	ChIP		rabbit

Secondary antibodies or conjugates

name	catalog no.	company	use	dilution	source
anti-mouse HRP	# W4021	Promega	WB	1:10.000	goat
anti-rabbit HRP	# A0545	Sigma-Aldrich	WB	1:10.000	goat
Cy3	ab6939	Abcam	IF	1:2000	goat
